# A genomic view of the NOD-like receptor family in teleost fish: identification of a novel NLR subfamily in zebrafish

**DOI:** 10.1186/1471-2148-8-42

**Published:** 2008-02-06

**Authors:** Kerry J Laing, Maureen K Purcell, James R Winton, John D Hansen

**Affiliations:** 1 Department of Pathobiology, University of Washington, Seattle Washington, 98195, USA; 2 US Geological Survey, Western Fisheries Research Center, Seattle, Washington, 98115, USA

## Abstract

**Background:**

A large multigene family of NOD-like receptor (NLR) molecules have been described in mammals and implicated in immunity and apoptosis. Little information, however, exists concerning this gene family in non-mammalian taxa. This current study, therefore, provides an in-depth investigation of this gene family in lower vertebrates including extensive phylogenetic comparison of zebrafish NLRs with orthologs in tetrapods, and analysis of their tissue-specific expression.

**Results:**

Three distinct NLR subfamilies were identified by mining genome databases of various non-mammalian vertebrates; the first subfamily (NLR-A) resembles mammalian NODs, the second (NLR-B) resembles mammalian NALPs, while the third (NLR-C) appears to be unique to teleost fish. In zebrafish, NLR-A and NLR-B subfamilies contain five and six genes respectively. The third subfamily is large, containing several hundred NLR-C genes, many of which are predicted to encode a C-terminal B30.2 domain. This subfamily most likely evolved from a NOD3-like molecule. Gene predictions for zebrafish NLRs were verified using sequence derived from ESTs or direct sequencing of cDNA. Reverse-transcriptase (RT)-PCR analysis confirmed expression of representative genes from each subfamily in selected tissues.

**Conclusion:**

Our findings confirm the presence of multiple NLR gene orthologs, which form a large multigene family in teleostei. Although the functional significance of the three major NLR subfamilies is unclear, we speculate that conservation and abundance of NLR molecules in all teleostei genomes, reflects an essential role in cellular control, apoptosis or immunity throughout bony fish.

## Background

In recent years, a family of molecules with roles in apoptosis and immune regulation has been discovered in mammalian genomes. This gene family, is known under several pseudonyms, including the CATERPILLER (CLR), NACHT, NOD-LRR or NOD-like receptor (NLR) family and is comprised of two major subfamilies of NOD and NALP molecules, along with 3 divergent members; IPAF, the MHC class II transactivator (CIITA) and neuronal apoptosis inhibitory protein (NAIP) [1,2]. Official names have been recently assigned to many members of this family by the HUGO Gene Nomenclature Committee (HGNC) [3] using the NLR prefix (Table 1). NLRs are recognized by the presence of three specific domains; an effector domain at the N-terminus that is involved in protein:protein interactions, a central NACHT (or nucleotide binding oligomerization/NOD) domain and a C-terminal leucine-rich repeat (LRR) domain. They are, therefore, structurally similar to disease resistance (R) proteins found in plants that are well known for their anti-microbial activities [4]. In humans, 22 NLRs have been described including 14 NALPs, with a PYRIN effector domain, and 5 NODs whose effector domain is typically a caspase recruitment domain (CARD).

**Table 1:** Human NLR sequences used for analyses

Nickname ^[1]^	HGNC approved symbol	NLR designation	CLR designation ^[2]^	Uniprot accession number
NOD1	NOD1	NLRC1	CLR7.1	[Swiss-prot:Q9Y239]
NOD2	NOD2	NLRC2	CLR16.3	[Swiss-prot:Q9HC29]
NOD3	NLRC3	NLRC3	CLR16.2	[Swiss-prot:Q7RTR2]
NOD4	NLRC5	NLRC5	CLR16.1	[Swiss-prot:Q86WI3]
NOD5	NLRX1	NLRX1	CLR11.3	[Swiss-prot:Q7RTR3]
NALP1	NLRP1	NLRP1	CLR17.1	[Swiss-prot:Q9C000]
NALP2	NLRP2	NLRP2	CLR19.9	[Swiss-prot:Q9NX02]
NALP3	NLRP3	NLRP3	CLR1.1	[Swiss-prot:Q96P20]
NALP4	NLRP4	NLRP4	CLR19.5	[Swiss-prot:Q96MN2]
NALP5	NLRP5	NLRP5	CLR19.8	[Swiss-prot:P59047]
NALP6	NLRP6	NLRP6	CLR11.4	[Swiss-prot:P59044]
NALP7	NLRP7	NLRP7	CLR19.4	[Swiss-prot:Q32MH8]
NALP8	NLRP8	NLRP8	CLR19.2	[Swiss-prot:Q86W28]
NALP9	NLRP9	NLRP9	CLR19.1	[Swiss-prot:Q86W27]
NALP10	NLRP10	NLRP10	CLR11.1	[Swiss-prot:Q86W26]
NALP11	NLRP11	NLRP11	CLR19.6	[Swiss-prot:P59045]
NALP12	NLRP12	NLRP12	CLR19.3	[Swiss-prot:P59046]
NALP13	NLRP13	NLRP13	CLR19.7	[Swiss-prot:Q86W25]
NALP14	NLRP14	NLRP14	CLR11.2	[Swiss-prot:Q86W24]
IPAF	NLRC4	NLRC4	CLR2.1	[Swiss-prot:Q9NPP4]
NAIP	NAIP	NLRB1	CLR5.1	[Swiss-prot:Q13075]
CIITA	CIITA	NLRA	none	[Swiss-prot:P33076]

The functions of the NLRs are presently not well defined. However, based on their structural characteristics, these molecules are thought to be expressed in the cytosol of immune-related cells, and have been implicated in autoimmune diseases and responses to bacterial [5] or viral molecules [6] supporting their importance in host immunity. Some of these molecules activate caspase-1 [7], while others initiate [8,9] or inhibit NF-κB signaling [10]. These two molecular pathways are fundamental to a molecular platform known as the inflammasome [7], which coordinates the production and processing of important inflammatory cytokines such as interleukin (IL)-1, IL-18 and IL-33 in mammals. Proteins that assemble in this caspase-1 inflammasome vary according to the cell type and stimulus [11]. Other molecules (e.g. caspase 11) that are necessary for inflammasome function are thought to be generated or recruited as a result of cross-talk between NLR and toll-like receptor (TLR) signaling [12]. According to current hypotheses, the activation of NLRs occurs following recognition of specific ligands by their LRR domains similar to the way that TLRs recognize molecules from extracellular pathogens. NLR proteins are, therefore, believed to represent cytosolic pattern recognition receptors (PRRs) that use LRR regions to detect intracellular pathogens. Those NLRs that are better defined functionally include NOD1, NOD2, and NALP3. NOD1 and NOD2, have both been shown to play a role in immunity of the mammalian gut and are highly expressed in epithelial cells or macrophages associated with the intestine. NOD1 recognizes a molecule known as meso-DAP (γ-D-glutamyl-meso-diaminopimelic acid), which is a peptidoglycan (PGN) component found in Gram-negative and Gram-positive bacteria [13], while NOD2 recognizes muramyl dipeptide, a peptidoglycan component found only in Gram-positive bacteria [14]. NALP3 (alias cryopyrin) has been shown to recognize a wide range of molecules, including bacterial RNA and synthetic viral RNA/DNA mimics (R837 and R848) [6]. NALP3 becomes activated in TLR-primed macrophages in response to ATP (adenosine triphosphate) and bacterial toxins that lower cytoplasmic K^+ ^[12], which is thought to be the major mechanism in the NALP3 response to certain Gram-positive bacteria. In a distinct pathway, monosodium urate and calcium pyrophosphate dihydrate crystals have been shown to increase caspase-1 activity in a NALP3-dependent (TLR-independent) manner [1], representing potential 'danger signal' ligands for NALP3 [5] and defining a further role for NLRs in recognizing cellular stress.

Members of the NLR family have not been extensively studied in taxa other than mammals, although recent reports indicate some members of this family exist in lower vertebrates [15] and in invertebrates [16]. Extending the knowledge of NLRs in ectotherms, this study reports an extensive overview of the NLR family in teleost fish, represented using information derived from the zebrafish *Danio rerio*. Here, we describe the gene phylogeny and expression of three major subfamilies of NLRs in teleostei, which we designate NLR-A and NLR-B (resembling mammalian NOD and NALP subfamilies respectively) and NLR-C, a large subfamily (characterized with a NOD-3-like NACHT domain and an unusual C-terminal domain) that appears in all teleostei genomes, and is unique to bony fish. The implications of all three subfamilies in immune regulation of fish are discussed.

## Results

**Figure 1:**
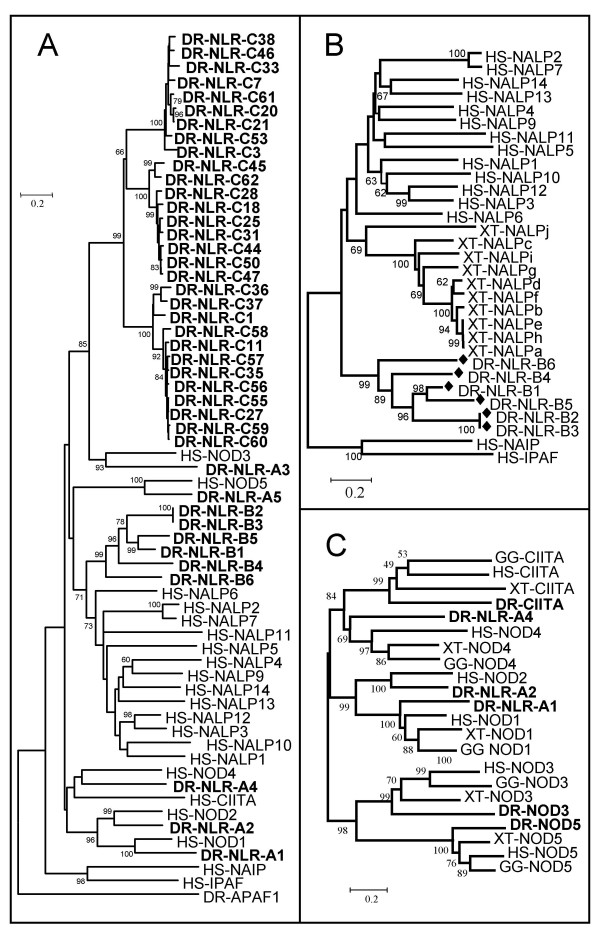
Phylogenetic comparison of vertebrate NLR molecules. Amino acid sequences of the NACHT domains (between the GxxGxGKS/T motif and the 'FAAFY' signature of human NOD2 or equivalent region in other NLRs) of vertebrate NLRs were aligned using CLUSTALW. Trees were constructed from these multiple alignments using the Maximum evolution and Neighbor-joining methods within the MEGA 3.1 program, using Poisson correction and complete deletion of gaps. Maximum evolution trees are shown. The resulting trees were bootstrapped 1000 times (shown as percentages). [A] Zebrafish NLRs were compared to human NLRs to estimate orthology. [B] The NALP subfamily was analyzed in more detail by comparing all zebrafish and *Xenopus tropicalis *predicted NALP-like molecules to human NALPs. [C] The NOD/NLR-A subfamilies of zebrafish, frog, chicken and humans were compared. DR = *Danio rerio*; XT = *Xenopus tropicalis*; GG = *Gallus gallus*; HS = *Homo sapiens*.

Many NLR-like sequences were identified in the genome and EST databases of non-mammalian vertebrates. These genes were compared by phylogenetic analysis of their deduced NACHT domains (Fig 1). Mammalian NLRs are categorized into NOD and NALP families according to previous publications [1] and as depicted in Table 1. In the zebrafish genome, three distinct subfamilies were identified and highly supported by bootstrap analysis; some resembled mammalian NODs (designated subfamily A; Table 2; Fig 1C), some resembled mammalian NALPs (designated subfamily B; Table 3; Fig 1B) and some formed a unique clade, closely related to NOD3, which was restricted to teleostei (designated subfamily C; Table 4; Fig 1A).

**Table 2:** NLR-SUBFAMILY A in zebrafish

Chromosome	Location (m)	Nickname	**ENSEMBL prediction ID **(ENSDARG000000)	REFSEQ/uniprot ID	**Domains identified**^a^
16	61.01	NLR-A1/NOD1	36308	XP_699379	C-N-L
7	47.25	NLR-A2/NOD2	10756	Q1AMZ9	C-C-N-L
24	41.14	NLR-A3/NOD3	61564	XP_697414	X-N-L
18	20.56	NLR-A4/NOD4	24632^c^		X-N-L
15	32.04^b^	NLR-A5/NOD5	89201	XP_685481	X-N-L

^a ^C = CARD, N = NACHT, L = LRR, X = unknown effector domain.

^b ^Two copies of NLR-A5 situated at 32.04 and 32.17 m on chromosome 15 are 100% identical and likely represent errors in the assembly of this chromosome.

^c ^NLR-A4 is also encoded within GENSCAN prediction GENSCAN00000002276.

**Table 3:** NLR-SUBFAMILY B in zebrafish

Chromosome	Location (m)	Nickname	**ENSEMBL prediction ID **(ENSDARG000000)	REFSEQ ID	Domains identified^a^
2	46.37	NLR-B1		XP_694951	N
	46.39	NLR-B2	38493	XP_694893	C-N
	48.19	NLR-B3	04582		N
	51.35	NLR-B4	38549		N
15	28.33	NLR-B5	56991		N
	28.49	NLR-B6	41744		N

^a ^C = CARD, N = NACHT

**Table 4:** NLR-SUBFAMILY C in zebrafish (selected examples)

Chromosome	Location (m)	Nickname	**ENSEMBL prediction ID **(ENSDARG000000)	REFSEQ ID	Domains identified ^a^
3	61.59	NLR-C1		XP_692844	X-N-L-B
	69.38	NLR-C2	52239		P-N
4	15.14	NLR-C3	57488	XP_688571	P-N-L
	37.15	NLR-C4	54997		P-N
	39.60	NLR-C5	54809		P-N
	39.94	NLR-C6	54769		P-N
	43.16	NLR-C7	54521	XP_689993	P-N-L
	43.36	NLR-C8	54506		P-N
	44.06	NLR-C9		XP_698595	
	44.97	NLR-C10	54412		N
	45.05	NLR-C11		XP_692726	X-N-L-B
	45.10	NLR-C12	54393		P-N
5	26.20	NLR-C13	56022		P-N
	45.15	NLR-C14	53289		P-N
6	39.57	NLR-C15	53299		P-N
7	83.86	NLR-C16	51797		P-N
8	18.11	NLR-C17	57564		P-N
9	48.99	NLR-C18		XP_686111	X-N-L
12	50.04	NLR-C19	52946		P-N
	54.00	NLR-C20	52760	XP_686816	P-N-L
	54.59	NLR-C21	52752	XP_689021	P-N-L
13	51.64	NLR-C22	52785		P-N
	51.86	NLR-C23	52776		P-N
14	37.23	NLR-C24	55661		P-N
	38.47	NLR-C25	55600	XP_693637	P-N-L
	38.57	NLR-C26	55597		P-N
	39.22	NLR-C27		XP_694145	X-N-L-B
	44.07	NLR-C28		XP_693652	X-N-L
	49.77	NLR-C29	54752		P-N
	52.05	NLR-C30	54465		P-N
	56.87	NLR-C31		XP_691478	N
	59.74	NLR-C32	53860		P-N
	60.05	NLR-C33	53848	XP_689023	P-N-L-B
	62.17	NLR-C34	53740		P-N
	69.67	NLR-C35	53051	XP_698503	P-N
17	8.51	NLR-C36		XP_690676	X-N-L-B
	9.80	NLR-C37		XP_689954	X-N-L-B
18	32.54	NLR-C38	56197	XP_693868	P-N-L
20	7.77	NLR-C39	43785		P-N
21	54.16	NLR-C40	52974		P-N
22	34.79	NLR-C41	55030		P-N
	35.49	NLR-C42	55004		P-N
	35.68	NLR-C43	54977		P-N
	47.49	NLR-C44		XP_688350	X-N-L-B
	96.65	NLR-C45		XP_697815	N
23	43.12	NLR-C46	54149	XP_687258	P-N-L
	44.18	NLR-C47		XP_690327	X-N-L-B
	47.00	NLR-C48	39900		N
Scaff 3690	480.02k	NLR-C49	53964		P-N
Scaff 3699	44.42k	NLR-C50		XP_687591	X-P-N-B
Scaff 3707	166.18k	NLR-C51	58788		P-N
Scaff 3711	372.30k	NLR-C52	55202		P-N
	638.24k	NLR-C53	55178	XP_691896	P-N-L-B
Scaff 3717	151.33k	NLR-C54	55023		P-N
Unknown		NLR-C55		XP_689864	X-N-L-B
Unknown		NLR-C56		XP_690860	X-N-L-B
Unknown		NLR-C57		XP_694605	X-N-L-B
Unknown		NLR-C58		XP_694871	X-N-L-B
Unknown		NLR-C59		XP_696432	X-N-L-B
Unknown		NLR-C60		XP_698814	X-N-L-B
Unknown		NLR-C61		XP_699128	P-N-L-B
Unknown		NLR-C62		XP_697396	N-L

^a ^P = PYRIN, N = NACHT, L = LRR, X = unknown effector domain

### Subfamily A

**Figure 2:**
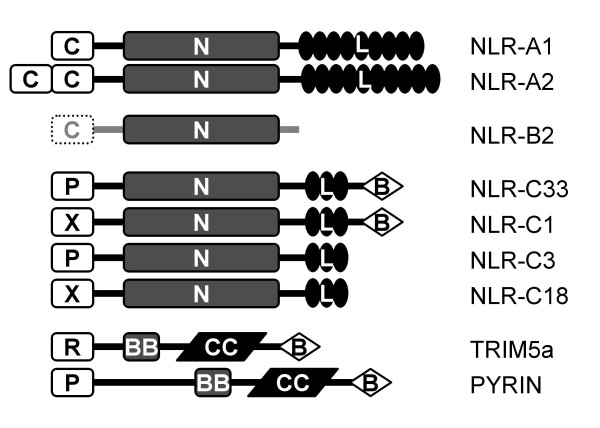
Schematic diagram depicting the deduced protein structures of zebrafish NLRs. Zebrafish NLR subfamily-A have structures similar to the NOD subfamily in mammals, with NOD1/NLR-A1 possessing one CARD motif while NOD2/NLR-A2 possesses two. While only the NACHT domain was identified for most members of the NLR-B subfamily, one member of this group (NLR-B2) was predicted with a putative N-terminal CARD effector domain. All NLR-C subfamily members were predicted to have an N-terminal effector domain, a central NACHT domain and a LRR domain. In addition, some of the NLR-C molecules were identified with a C-terminal B30.2 domain. The predicted effector domains of molecules within the NLR-C subfamily varied; some had a pyrin (P) effector domain, while others had a distinct uncharacterized effector domain (X). B30.2 domains are also found in other important immune related molecules such as certain TRIMs and the Pyrin molecule, whose structures are also shown. C = card domain, P = pyrin domain, X = other domain, N = NACHT domain, L = LRR region, B = B30.2/PRY-SPRY domain, R = ring finger domain, BB = B-box, CC = coiled coil.

Teleost fish possess gene orthologs for all five members of the mammalian NOD subfamily (Table 2; Table 5). While chicken and *Xenopus *genomes apparently lack the NOD2 gene (Table 5; Fig 1C), both are in possession of the remaining four NODs. The gene predictions for zebrafish NOD sequences were corrected using corresponding ESTs identified in the TIGR database, and missing sequence found with assistance from other fish NOD-like sequences using the BLAT (BLAST-like alignment tool) program. Following assembly, zebrafish NODs were highly structurally conserved relative to human NODs. Zebrafish NOD1 (NLR-A1) has an N-terminal CARD domain, and nine highly conserved leucine-rich repeats (LRRs) (Fig 2) although the 5' and 3' exons were not identified. Two CARD domains were identified at the N-terminal end of zebrafish NOD2 (NLR-A2), and eight LRR domains were recognizable by their LRR-like motifs (e.g. the amino acid signature LxxLxLxxCxL, where L = L, I, V or F and C = C or N) that align exactly with the LRRs of human NOD2 [see Additional file 1]. Although the N-terminal end of NOD3 (NLR-A3) was not recognized by the CDD, it shares some similarity with the human NOD3 effector domain with two conserved sequence signatures, MRK and EAG (amino acids 59–61 and 69–71 of DR-NLR-A3 respectively). The C-terminal end of NLR-A3 possessed 14 LRR motifs, which aligned exactly with LRR domains of human NOD3 [see Additional file 1], with a similar motif (CxxLxMxxNxF) between the NACHT domain and the first true LRR motif. Both NOD4 (NLR-A4) and NOD5 (NLR-A5) orthologs in zebrafish have conserved sequences within their LRR domains relative to their human equivalents, although the predicted LRR for zebrafish NLR-A4 is shorter than that of human NOD4, and less conserved relative other human and zebrafish NLR-A orthologs. The N-terminal domains for NLR-A4 and NLR-A5, as with NLR-A3, were not identified within the CDD database set, but share some conserved features with the corresponding regions of mammalian NOD4 and NOD5. NLR-A4/NOD4 appears to represent the most divergent gene within the NLR-A subfamily, yet zebrafish NLR-A4 groups with high bootstrap support with human NOD4 and its orthologs from chicken and *Xenopus *during phylogenetic comparisons (Fig 1C). All five NLR-As are located on distinct chromosomes in zebrafish. The NLR-A1 gene is located on chromosome 16 in version 6 of the zebrafish genome (Zv6) (Table 2), but is not mapped to a chromosome in version 7 (Zv7) [see Additional file 2], NLR-A2 resides at chromosome 7 and NLR-A3, NLR-A4 and NLR-A5 can be found on chromosomes 24, 18 and 15 respectively.

**Table 5:** NLR subfamily genes identified in other non-mammalian genomes

Genome	Chromosome	Nickname	Closest ortholog	Identifier	Effector domain
*Gallus gallus*	Chr 2	GG-NOD1	NOD1	ENSGALG00000011535	CARD^a^
	Chr 14	GG-NOD3	NOD3	XM_414969	CARD^b^
	Chr 11	GG-NOD4	NOD4	XM_414012	?
	Chr 24	GG-NOD5	NOD5	XM_417893	?
	Chr 5	GG-NALP	NALP3	Q5F3J4	PYRIN
*Xenopus tropicalis*	Scaff 320	XT-NOD1	NOD1	ENSXETG00000022012	CARD^a^
	Scaff 27	XT-NOD3	NOD3	GENSCAN00000015124	CARD^b^
	Scaff 112	XT-NOD4	NOD4	AC157698	?
	Scaff 124	XT-NOD5	NOD5	ENSXETG00000021586	?
	Scaff 279	XT-NALPa	NALP6	scaffold_279.46^c^	?
	Scaff 1247	XT-NALPb	NALP6	scaffold_1247.5	?
	Scaff 3599	XT-NALPc	NALP6	scaffold_3599.1	?
	Scaff 626	XT-NALPd	NALP6	scaffold_626.23	?
	Scaff 279	XT-NALPe	NALP6	scaffold_279.46^c^	?
	Scaff 626	XT-NALPf	NALP6	scaffold_626.24	?
	Scaff 626	XT-NALPg	NALP6	scaffold_626.25	?
	Scaff 279	XT-NALPh	NALP6	scaffold_279.46^c^	?
	Scaff 279	XT-NALPi	NALP6	scaffold_279.46^c^	?
	Scaff 111	XT-NALPj	NALP6	scaffold_111.48	?
	Scaff_74	XT-IPAF	IPAF	ENSXETG00000018490	CARD
*Fugu rubripes (v4)*	Scaff 58	FR-NOD1	NLR7.1	NEWSINFRUG00000144256	CARD^a^
	Scaff 28	FR-NOD2	NLR16.3	NEWSINFRUG00000134206	CARD^b^
	Scaff 3	FR-NOD3	NLR16.2	NEWSINFRUG00000138086	CARD^b^
	Scaff 140	FR-NOD4	NLR16.1	GENSCANSLICE00000000561	?
	Scaff 144	FR-NOD5	NLR11.3	NEWSINFRUG00000148012	?

^a ^Identified using the Conserved Domain Database,

^b ^Based on similarity to human ortholog,

^c ^Xenopus gene prediction scaffold_279.46 contains NACHT domains from multiple NLRs.

### Subfamily B

Six distinct genes encoding NACHT domains were identified in Zv6 that belong to subfamily B and form a separate cluster within the clade of mammalian NALPs. Although zebrafish NLR-B2 and B3 were identified in distinct regions of the zebrafish genome (Table 3) these genes are identical in the region of the NACHT domain used for phylogenetic analysis. Several NALP-like sequences were also identified for *Xenopus tropicalis *(Table 5) that similarly formed their own cluster distinct from the human and zebrafish NALPs (Fig 1B). Gene predictions encoding putative NALPs in zebrafish are short, with most lacking a recognizable effector domain and C-terminal LRR domain. One exception is NLR-B2, which appears to have an N-terminal region with low similarity to a CARD motif as identified by searching the CDD. Only one cDNA sequence resembling this subfamily could be identified in the zebrafish EST database [GenBank:AI883819] that, although highly similar in sequence, was not an exact match to any of the predicted NLR-B genes and appeared to encode only a portion of the NACHT domain. NLR subfamily B genes appear to be restricted to small clusters on chromosomes 2 and 15 in zebrafish. Furthermore, NLR-B5 and NLR-B6 reside close (28.3–28.5 m) to NLR-A5 (32 m) on chromosome 15. Later analysis of Zv7 revealed removal of the NLR-B5 gene prediction, and its merger with the prediction for NLR-B6 [see Additional file 2].

### Subfamily C

Database searches revealed multiple genes that possessed NACHT domains and shared significant homology to human NOD3 yet were distinct from the zebrafish NOD3 molecule (NLR-A3) described above. This large number of highly similar genes clearly arose from several gene(ome) duplication events. Several hundred predicted genes/proteins were observed for this group in the databases for all teleost fish (data not shown). In zebrafish, these genes were found at numerous chromosomal loci, with large clusters evident on (at least) chromosomes 1, 4, 14 and 17. A small selection of these genes was subjected to further analysis (Table 4 and Fig 1A). These molecules divided into three clusters during phylogenetic analysis, which also corresponded to sequence differences identified in the N-terminal region. Representatives from chromosome 14 were identified in all three clusters, while NLRs from some other chromosomes (e.g. chromosomes 12 and 17) were restricted to one cluster, although not all genes were included in the analysis.

**Figure 3:**
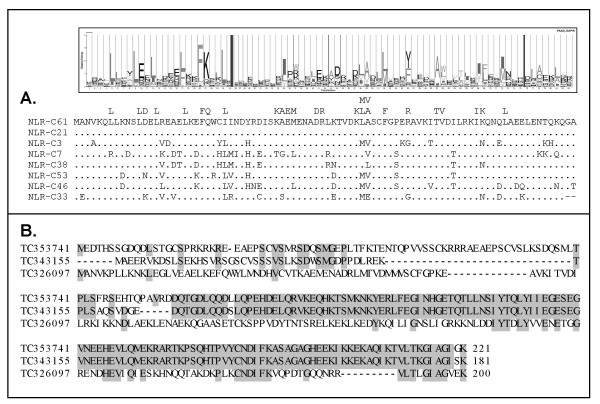
Many N-terminal effector domains of the predicted zebrafish NLR-C molecules are recognized as pyrin/PAAD-DAPIN domains based on the HMM logo. Some examples are shown, with conserved amino acids [A]. Other N-terminal sequences were observed for NLR-C molecules, which were confirmed in the EST databases at TIGR [B]. TC326097 encodes a pyrin domain, whereas TC353741 and TC343155 represent undefined N-terminal domains such as those denoted by 'X' in Figure 2.

**Figure 4:**
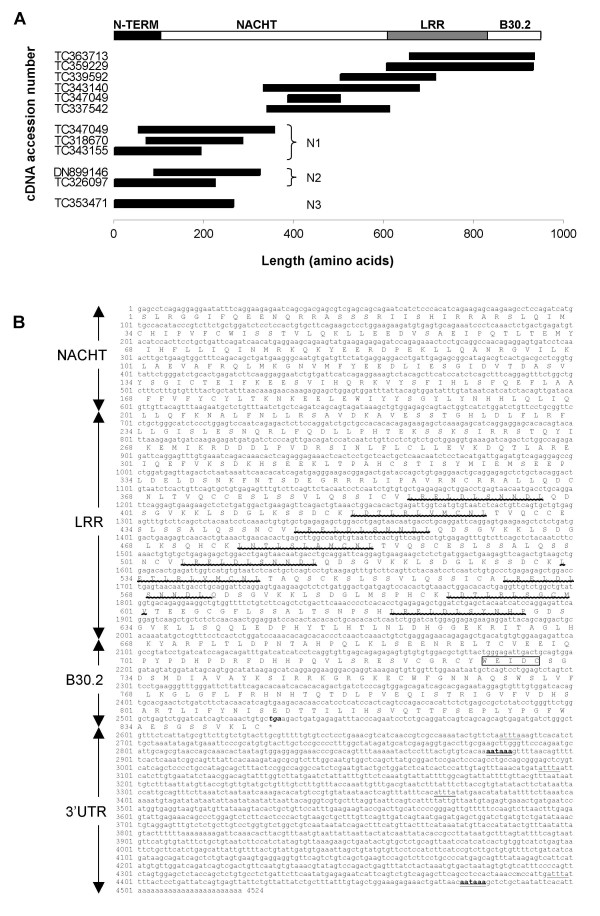
A. Schematic representation of the approximate positions of NLR encoding EST sequences relative to predicted NLR-C proteins. TIGR database accession numbers for the ESTs are given. N1 = X effector domain beginning with sequence MAEERV, N2 = recognized Pyrin effector domain, N3 = X effector domain beginning with sequence MEDTHS. B. Full cDNA sequence for EST CK126487 that spans a region from the NACHT domain to the 3'UTR of an NLR-C gene. LRR signatures are indicated with a wavy underline, and the signature for the B30.2 domain is boxed. Features for polyadenlyation (AATAAA) and mRNA instability (ATTTA) in the 3'UTR are double or single underlined respectively, and the stop codon (tga) is shown in bold italics.

**Figure 5:**
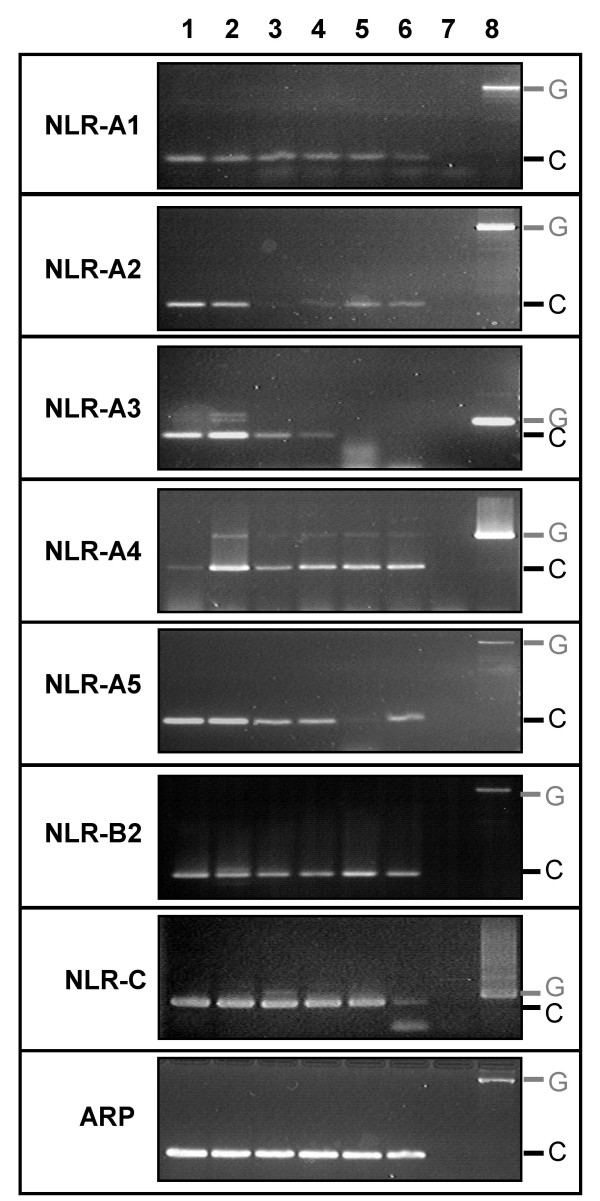
RT-PCR analysis of the NLR gene family in intestine (1 and 2), liver (3 and 4) and spleen (5 and 6) of two individual naïve zebrafish. ARP expression was amplified to verify cDNA synthesis. Negative controls were performed using templates from cDNA synthesis reactions without reverse transcriptase (7). Genomic DNA (8) was amplified to verify primer efficiency and to show the difference in size of genomic amplicons compared to cDNA amplicons.

Although the NACHT domains of the C-group NLRs are clearly homologous to NOD3, many of these genes were found to encode a conserved PYRIN domain at the N-terminus (Figs 2, 3) showing some analogy to mammalian NALP genes. The presence of this domain was confirmed by identifying an EST sequence containing the PYRIN domain and a partial NACHT domain that resembled C-group NLRs. Other C-group NLRs had N-terminal sequences with no obvious gene ortholog. While some are likely incorrectly predicted domains, EST sequences confirm at least two of these predicted N-termini are transcribed in association with the NLR C-group NACHT domain (Fig 4). The NLR C-group molecules also possess an LRR region, as with other NLRs. Unexpectedly, a B30.2 (PRY-SPRY) domain was identified in several of the predicted genes for NLRs of the C subfamily. Owing to nature of this large multigene family, a single representative EST was sequenced to confirm domain structure, including the verification of the B30.2 domain. The B30.2. This domain was found at the C-terminus, following the LRR domain and was confirmed by completely sequencing EST [Genbank:CK126487]; the 4,524 bp sequence was submitted to GenBank [GenBank:EF613347] and contained sequence from the NACHT domain to the poly-A tail. Further overlapping ESTs/TCs identified in the TIGR database provided additional confirmation for the NLR C-group, with an effector (PYRIN or other) domain, NACHT domain, LRR domain and a C-terminal B30.2 domain (Fig 5) [see Additional file 3] although, due to the high number of closely related sequences for this subfamily and the current stage of the zebrafish genome sequence, it was not possible to ascertain whether the overlapping ESTs were generated from the same gene or distinct genes within the NLR-C family.

### Other NLRs

Although the CIITA was evident in the genomes of the pufferfishes, frog and chicken, this molecule was not readily identifiable in zebrafish Zv6. However, later analyses identified a CIITA-like gene, in Zv7 of the zebrafish genome, which resides on Chromosome 3 at approximate position 24.3 m (Zv7_scaffold 244). Sequences for NAIP were not identified in lower vertebrates during this study. IPAF was identified in the frog genome (Table 5), but not in the other non-mammalian genomes. A recently described family of NLR-like genes from the sea urchin was found to cluster with mammalian IPAF and NAIP molecules during phylogenetic analyses (data not shown).

### Expression of zebrafish NLRs

The spatial expression of NLR genes was evaluated in selected tissues from naïve zebrafish. NLR-A1, -A2, -A3 -A4 and -A5 were all identified in zebrafish intestine using RT-PCR. All five genes were also expressed in liver although expression of NLR-A2 was extremely weak. NLR-A3 expression was not detected in the spleen following 35 PCR cycles, but the four other NLR-A genes were expressed in this tissue (with low expression of splenic NLR-A5 in one individual). As a representative of the NLR-B subfamily, NLR-B2 expression was investigated and detected in all three tissues. Similarly, mRNA was detected for an NLR-C gene(s) in all three tissues using primers based on the completely sequenced EST clone. The primers used to detect these genes amplified no products in control reactions whose templates were sterile water (not shown) or from cDNA syntheses performed in the absence of reverse transcriptase (RT-). ARP was amplified from all tissues confirming the integrity of the cDNAs and the success of RT-PCR. Genomic products were amplified for all NLR-A genes that were larger than the cDNA amplicons and supported the presence of intron(s) between the primer regions.

## Discussion

New insight into the regulation of essential developmental, inflammatory and apoptotic pathways was achieved with the discovery and characterization of the NLR gene family of putative cytoplasmic pattern recognition molecules. While an increasing amount of information exists for these molecules in mammals, this gene family is poorly studied in other vertebrates with little to no information available even at the gene level for birds, amphibians and fish. This study resolves this issue by identifying and characterizing many NLR-like genes from these three classes of animals and uncovering a unique subfamily of NLRs in teleost fish.

Our evidence shows early evolution and high conservation of the NOD (NLR-A) subfamily of NLRs. All species of teleost fish that were analyzed had five distinct members of this subfamily designated NLR-A1, NLR-A2, NLR-A3, NLR-A4 and NLR-A5 that were clear gene orthologs of human NOD1 to NOD5 [1]. A NOD1 ortholog was also described during an earlier screen of zebrafish ESTs for molecules similar to apoptosis regulators [17]. In addition to encoded NACHT domains, the effector domains and LRR regions were highly conserved in the fish NLR-A genes relative to their human equivalents, suggesting retained function. NLR-A1 and NLR-A2, the fish orthologs of human NOD1 and NOD2 respectively, both possessed clear CARD domains (one in NLR-A1 and two in NLR-A2) with high amino acid identity to the equivalent regions of human molecules. In mammalian NOD1 (and presumably NOD2), the CARD domains are necessary for the interaction with RICK kinase, an enzyme that participates in NFκB activation and, ultimately, the generation of pro-inflammatory molecules [18]. Since RICK is also present in fish genomes (see zebrafish RIPK2, Q4V958), it would appear that this inflammatory cascade was established prior to the divergence of teleost fish from the tetrapod lineage, assuming that the same interaction occurs between these molecules in fish. The highly conserved sequences in the LRR domains implies these zebrafish NLR-A1 and NLR-A2 may also be able to recognize meso-DAP and muramyl dipeptide as mammalian NOD1 and NOD2 respectively [13,14] although this requires formal confirmation. NLR-A1 transcript was detected equally in intestine, liver and spleen, reflecting the wide-spread distribution observed for murine NOD1 [18], while NLR-A2 was strongly expressed in intestine, with some expression in spleen and barely detectable levels in liver. Similar to the highest expression of NLR-A2 in zebrafish intestine, human NOD2 has a more restricted expression pattern, with predominant expression in cells of myeloid origin including monocytes [9] and Paneth cells [19] that are associated with the gut, although expression of NOD2 can also be induced in epithelial cells [20]. Zebrafish NLR-A3 is clearly an ortholog of mammalian NOD3, with similarity in the effector and NACHT domains and an equal number of LRR domains. At the genomic level, NOD3 is flanked by RHOT2, SBK1 and PDPK1, GNPTG respectively in zebrafish and fugu further supporting the orthologous relationship for NOD3 between fish species. Expression of NLR-A3 was strong in zebrafish intestine, with some expression also in liver and little to no expression observed in the spleen. Interestingly, the kidney (bone marrow equivalent) did not express NLR-A3 as well suggesting that it is not expressed by lymphocytes (data not shown). In mammals, NOD3 expression occurs primarily in lymphocytes and is attributed to inhibition of T-cell activity [21]. Two other NLR-A subfamily members were also identified in zebrafish that were designated NLR-A4 and NLR-A5 with NLR-A4 resembling human NOD4 and NLR-A5 being highly conserved to human NOD5. NLR-A4/NOD4 genes represent the most divergent members of this subfamily based on amino acid conservation within the N-terminal and LRR regions between different vertebrate orthologs. Both NLR-A4 and NLR-A5 genes were constitutively expressed in intestine, spleen and liver of naïve zebrafish, although there is clearly some fish to fish variation preventing their detection in some individuals under the conditions used for RT-PCR. Currently, there is no information concerning the expression patterns or functions of these latter two NLRs in mammals.

Whereas NOD1, NOD3, NOD4 and NOD5 appear to be conserved in bird and amphibian genomes, the gene for NOD2 was identified in neither the chicken nor the frog genomes. This would suggest that NOD2 has been deleted from the genomes in these species, although the genome of *Xenopus tropicalis *is, at present, incomplete. This is surprising since NOD2, in mammals, appears to be a highly important sensor for intracellular microbial molecules. However, chickens do possess a NALP3 ortholog (see below) representing another potential PRR for muramyl dipeptide [22] and may functionally replace NOD2 in this species.

Members of the NALP subfamily are also evident in lower vertebrates. Six genes were identified in zebrafish (Zv6) for NALP-like molecules (*NLR-B1 *to *-B6*), and ten predicted NALP-like genes (nicknamed *NALPa *to *NALPj*) were found for *Xenopus*. These genes clustered separately for each species, suggesting recent duplication events formed the NALP subfamilies independently in fish, amphibians and mammals. The closest human ortholog of the amphibian and fish NALPs appears to be NALP6. A single NALP-like sequence predicted in the chicken genome (ENSGALG00000005155) and in the Uniprot database (Q5F3J4) clusters closest to the group of human NALPs 1, 3, 10, and 12 when analyzed phylogenetically (although not with strong bootstrap support) and has recently been given the name NLRP3 (previously designated CIAS1/NALP3). Chicken NALP was identified on chromosome 5, separate to the chicken *NOD5 *gene (chromosome 24). Although sequence variation makes accurate comparisons difficult, it is likely that this chicken gene arose from a distinct NALP than the fish and amphibian NALPs, with the ancestral NALP(s) possibly lost from the genome. The discovery of multiple NALP-like proteins in lower vertebrates contradicts a recent hypothesis by Hughes suggesting that the NALP subfamily evolved only in mammals [15], with clear evidence that a gene encoding the NACHT domain of at least one NALP (possibly a NALP6-like gene) was present prior to the fish-tetrapod split. Zebrafish NALPs are situated at two distinct chromosomal locations, four of these genes (*NLR-B1 *to -*B4*) are located on chromosome 2, and the other two (*NLR-B5 *and *-B6*) can be found near *NLR-A5 *on chromosome 15; the new assembly of the zebrafish genome (Zv7) suggests these two sequences may represent the same gene. It should be pointed out that although chicken NLRP3 has an N-terminal PYRIN domain, the N-terminal domains for the *Xenopus *and zebrafish NALPs were not identified. One exception was NLR-B2, which appears to have a domain that resembles a CARD and not a PYRIN domain as would be expected from its similarity to the mammalian NALPs. No PYRIN domains are observed for the *Xenopus *NALP-like sequences and, other than the PY-CARD protein (prediction ENSXETT00000004042), no PYRIN domains were predicted in the *Xenopus *genome. These observations may reflect that early ancestors of NALPs lacked these effector domains and later acquired the PYRIN domain (or CARD domain in the case of NLR-B1). Whether these NALP-like genes encode functional PRRs in poikilotherms remains uncertain, however, NLR-B2 transcript was detectable in zebrafish intestine, spleen and liver suggesting this may represent a functional gene.

In addition to the NOD- and NALP-like subfamilies, a unique subfamily of NLRs was identified in teleost fish, and designated NLR subfamily C (NLR-C). This subfamily is interesting for several reasons. Firstly, all teleostei genome (and EST) databases show numerous NLR-C genes, amounting to several hundred of these genes in a single species. Secondly, these genes all possess a central NACHT domain that is highly similar to the NACHT domain of NOD3 (NLR-A3) suggesting they evolved from a NLR-A3-like molecule, yet many of these genes possess a PYRIN domain at their N-terminus making them more structurally similar to mammalian NALP molecules. Finally, following the LRR domain many of these molecules (representatives found in *all *bony fish) possess a B30.2 (PRY-SPRY) domain, which may allow them to interact with distinct molecules to standard NLRs and thus perform some novel function. B30.2 domains are also found on some tripartite motif containing (TRIM) proteins [23] and on the PYRIN molecule [24] (Fig 2) and have several roles related to immunity. TRIM5a has been shown to inhibit retroviral activity by directly binding the capsid of the HIV retrovirus [25], and PYRIN has been shown to inhibit the activity of caspase-1 by directly binding to the active site of this enzyme [24], both using their B30.2 domains for these interactions. Each of these functions would fit with the role of NLRs as intracellular PRRs; the ability to bind viruses could be an extension of the pattern detection system attributed to the neighboring LRR domain, while the potential to inhibit caspase-1 activity may make NLR-C molecules important negative regulators of the inflammasome in teleost fish. The latter function would reflect gene families of cell surface receptors such as killer immunoglobulin-like receptors (KIRs) [26] or novel immune-type receptors (NITRs) [27] that possess many inhibitory receptors and a small number of stimulatory receptors for controlling cellular activation. It is also interesting that these molecules all contain a NACHT domain similar to NOD3, since mammalian NOD3 has an inhibitory role in T cells [21]. Importantly, since the predicted N- and C-termini of some NLR-Cs are structurally similar to the two domains of the PYRIN molecule, this would also fit with a potential function of mimicking PYRIN. However, additional studies are required to determine what, if any, role in the immune system NLR-C molecules may play.

The evolutionary processes generating the vast subfamily of NLR-C genes are not clear and appear very complex. The relationships are further confused by apparent errors in the assembly of the zebrafish genome (Zv6 versus Zv7), as evidenced by clear differences in the mapping of some NLR-C genes to their predicted chromosomes between assembly versions [see Additional file 2]. However, evidence suggesting tandem duplications of individual genes within a chromosomal locus is consistent between Zv6 and Zv7, which result in NLR-C genes adopting new exons encoding distinct N-terminal domains and/or C-terminal domains via exon-shuffling. The clusters of tandem NLR-C genes appear to have undergone *en bloc *duplication, to generate further clusters in the same locus (*cis *duplication), or within distinct loci or chromosomes (*trans *duplication) through translocation. Single genes may also have duplicated independently multiple times, within established loci and to create new loci, prior to and following formation of new gene structures. A large scale duplication of this gene family may be explained by the teleost-specific genome duplication event (3R) occurring early in the evolution of teleost fish, which followed two rounds of complete genome duplication (2R) observed early in the evolution of the vertebrate lineage [28]. Should this be the case, mutations and deletions of many of the duplicated genes would be expected, to remove redundancy from the genome [29]. Therefore, many NLR-C genes may be non-functional genes or pseudogenes, although a small number have likely established new functions. Clearly, it is too early to assign functionality to these genes, except to note that many are transcribed and are presumably translated into protein products. Transcripts for NLR-C were detected, in this study, in three distinct tissues in naïve zebrafish, and many more can be identified in the EST databases for this fish species.

A CIITA-like gene was identified in the zebrafish genome (Zv7) and is an important molecule, in mammals, for controlling the expression of both major histocompatibility complex class I and class II molecules and therefore is significant for antigen presentation to T lymphocytes. Defects in human CIITA gene expression have been linked to several immune disorders [5]. However, alternative molecules have been implicated in the induction of antigen presentation pathways [30], including other members of the NLR family, such as NALP12 [31]. NAIP/IPAF homologs have been identified in the sea urchin [16] implying that the ancestral NLR resembled one of these molecules. However, neither NAIP nor IPAF was identified in the fish genomes at this time, although IPAF was evident in the frog genome, suggesting that the genes for these molecules may have been lost from the fish genomes during the teleost-specific genome duplication event.

## Conclusion

In summary, the NLR gene family contains several members in all vertebrates, and at least one prototypical gene must have existed prior to the evolution of vertebrates. Clearly, there are some losses and gains of NLR genes in the genomes of distinct species thus shaping unique repertoires of these molecules throughout vertebrates and invertebrates. Although there are still many members of the NLR family that require functional characterization, their implication as regulators of immunity is highly intriguing and warrants future investigation.

## Methods

### Identification of NLRs in non-mammalian vertebrates

The amino acid sequences for human NLRs were obtained from UNIPROT (Release 9.0) [32]. These are listed in Table 1, with recently defined nomenclature assigned at HGNC [3]. Predicted genes for non-mammalian NLRs were identified in the UNIPROT database and at ENSEMBL [33] for chicken *Gallus gallus*, pipid frog *Xenopus tropicalis*, Japanese pufferfish *Fugu rubripes*, green spotted pufferfish *Tetraodon nigroviridis *and zebrafish *Danio rerio*, by using the BLAST algorithm to search for sequences with similarity to human NLRs [34]. Gene predictions were also identified in ENSEMBL by keyword searches for NACHT, PYRIN or CARD domains. EST sequences for these species were identified by BLAST-based searching the TIGR gene indices [35] or the "other vertebrate EST" section of GenBank at NCBI, and used to confirm and correct the gene predictions. The unique NACHT-LRR-B30.2 arrangement for NLR-C was determined by completely sequencing zebrafish EST CK126487 [GenBank:EF613347]. The EST was obtained from the American Type Culture Collection (ATCC) (Image number 7049223) and sequencing was carried out in-house using an ABI 3030 automated sequencer, universal (SP6/T7) and gene specific primers (located in Table 6) and BigDye V3.1.

**Table 6:** Oligonucleotide primers used for RT-PCR analyses and sequencing

Target	Primer name	Sequence 5'-3'
NLR-A1	ZF-NOD1ex-F1	TTA ACG ATT ATG GCG TGA AGC
	ZF-NOD1ex-R1	GAA CCT CAA TAC CGC TGT CTG
NLR-A2	ZF-NOD2ex-F1	TGG ATG TTG AGC ACC TGA AG
	ZF-NOD2ex-R1	CCC TTT TCC AGA AGT TTT CG
NLR-A3	ZF-NOD3ex-F1	TCT GTG CTG TTC TCG CCT ATT
	ZF-NOD3ex-R1	ACT CAG CAG ACT TCC CAA CAG
NLR-A4	ZF-NOD4ex-F1	AAA CTG GCA AAG GCT ATT GGT
	ZF-NOD4ex-R1	CAG GAT ACC GCA CAA AGT CTC
NLR-A5	ZF-NOD5ex-F1	TGA ATG AGC TGA ACC TGT CCT
	ZF-NOD5ex-R1	TCA GCC GCT GTA GAG AGT GAT
NLR-B2	NLRB FI	CAG ATC AAA GTG TGC AGC AGG TTC
	NLRB RI	TTG TGG AAC GAC TCT GTG AAG CTC
NLR-C	NLRC-F	GAA GAG GAT TAC AGC AGG ACT
	NLRC-R	TCT CCA CTG CAG TCA ATC TC
ARP	ZF ARP 35F	TTA AAC CGG CTG TTC ACC GAT CCT
	ZF ARP 271R	CGA ATG GCC TTC CTC ATC ATG GTG TT
EST-CK126487	ZF-EST487-F1	GTC TAC AGC TTC ATC CAT CT
	ZF-EST487-R1	ACA GAC TGA ATG ATT CTC TCA
	ZF-EST487-F2	AGC TCT GCT ACA GGA CTG T
	ZF-EST487-R2	CTC ATT ACT GAT CAA CTG AAC A
	ZF-EST487-F3	CCT CAA TCT GTG CCC T
	ZF-EST487-R3	CGG AGT AAA GCT GCT A

Chromosomal locations for the NLRs were deduced by matching the translated NLR sequences against the genomes using BLAT [36] at the UCSC Genome Browser database [37]. Specific domains within the zebrafish NLRs were confirmed by searching the Conserved domain database (CDD v 2.09) [38] at NCBI, by comparison to the PFAM hidden Markov Model (HMM) logos [39] and by direct comparison to putative mammalian orthologs. Genome versions used during these analyses are *G. gallus *assembly version 2.1 (May 2006), *X. tropicalis *assembly version 4.1 (August 2005), *T. nigroviridis *assembly version 7 (February 2004), *T. rubripes *assembly version 3 (August 2002) in BLAT and version 4 (December 2005) in ENSEMBL, and *D. rerio *assembly version 6 (March 2006). Following submission of this manuscript, assembly version 7 of the zebrafish genome became available, and all gene predictions were reanalyzed against this assembly. Data from Zv7 are available in supplementary tables [see Additional file 2].

### Phylogeny of NLRs

The phylogenetic relationships between zebrafish NLRs and human NLRs were predicted using both the maximum evolution and neighbor-joining methods within the MEGA 3.1 program [40]. Partial amino acid sequences from the NACHT domain (from regions corresponding to the GxxGxGKS motif to the FAAFY sequence signature of human NOD2) were used in the analyses as this region was clearly identified in all NLRs. Further analysis of the NLR-A and NLR-B subfamilies including frog and chicken NLRs were performed using the same methods. All trees were constructed from CLUSTALW generated alignments [41], using Poisson correction, complete deletion of gaps, and bootstrapped 1000 times.

### Expression of zebrafish NLRs

The specific expression patterns of the NLR gene family in zebrafish tissues were investigated. Zebrafish (Ekwill strain) were obtained from Ekwill Fish Farm, FL and reared in sand-filtered and UV-treated freshwater at a constant temperature of 24°C. Fish were fed a daily ration of adult zebrafish diet (Zeigler). Genomic DNA was extracted from fin tissue using the DNeasy extraction kit (Qiagen) following manufacturer's instructions. The spleen, liver and intestinal tissues were removed from two individuals and RNA was extracted using the RNeasy RNA extraction kit with in-column DNAse treatment (Qiagen) following manufacturer's instructions. Total RNA was purified and cDNA was synthesized as previously described [42]. A control, containing liver RNA but lacking reverse transcriptase, was also synthesized. The 20 μL cDNA synthesis reactions were diluted to a final volume 100 μL and stored at -20°C until use. PCR amplifications were performed in a 25 μl final reaction volume containing 2 μL of diluted cDNA, reagents from the *Taq *core PCR kit (Qiagen) and 12.5 pM of each primer. Primer pairs used to detect transcripts for each NLR gene are listed in Table 6 with their sequences. Cycling conditions for all amplifications consisted of 95°C for 3 min, 35 cycles of 94°C for 30 sec, 55°C for 30 sec and 72°C for 1 min, followed by final extension of 10 min at 72°C. Amplified products were subjected to electrophoresis on a 3% agarose gel and visualized by ethidium bromide staining.

## Abbreviations

BLAST: Basic local alignment search tool; BLAT: The BLAST-like Alignment Tool; CDD: Conserved Domain Database; EST: Expressed sequence tag; HMM: Hidden Markov model; meso-DAP: γ-D-glutamyl-meso-diaminopimelic acid; PRR: pattern recognition receptor; RT-PCR: reverse transcriptase-PCR; TIGR: The Institute for Genome Research.

List of proteins and protein family names:

CATERPILLER: CARD, Transcription Enhancer, R (purine)-binding, Pyrin, Lots of Leucine Repeats; CLR: CATERPILLER-like receptor; CIITA: MHC class II transactivator; IL: interleukin; IPAF: Ice protease-activating factor; MHC: Major histocompatibility complex; NAIP: neuronal apoptosis inhibitory protein; NALP: Nacht Domain-, Leucine-Rich Repeat-, and PYD-Containing Protein; NF-κB: Nuclear factor kappa b; NLR: NOD-like receptor; RICK: RIP-like-interacting CLARP kinase; TLR: Toll-like receptor.

List of conserved domain names:

CARD: caspase recruitment domain; LRR: Leucine-rich repeat; NACHT: NAIP, CIITA, HET-E (bacterial nucleotide triphosphatase protein) and TP1 (telomerase-associated protein); NOD: nucleotide oligomerization domain; SPRY: SPla/RYanodine receptor; TRIM: tripartite motif-containing.

## Authors' contributions

KJL carried out data mining, phylogenetics and bioinformatics analyses, participated in the conception, design and coordination of the study, and drafted the manuscript. MKP designed and performed the expression analyses, participated in the conception, design and coordination of the study, and assisted in the writing and approval of the manuscript. JRW provided advice and gave final approval for publication. JDH performed in-house sequencing of EST clones, assisted in primer design, provided advice, read, edited and approved the final manuscript.

## Supplementary Material

Additional File 1 Comparison of human and zebrafish NLR-A molecules. Alignment of predicted translations of zebrafish NLR-A genes with their predicted human orthologs. Sequence signatures in the NACHT domains used as boundaries for phylogenetic analyses are highlighted in yellow, and putative leucine-rich repeat motifs (or variants) are highlighted in red. Conserved amino acids between sequences are shown with "*", while ":" and "." represent high or low similarity of amino acids respectively. Click here for file

Additional File 2 Tables describing gene predictions and their locations for NLR-A, -B and -C genes in zebrafish genome assembly version 7. Click here for file

Additional File 3 The B30.2 domain of zebrafish NLR-C. Alignment of zebrafish NLR-C57 (see Table 4) with similar cDNA sequences identified in the TIGR database. The B30.2 (PRY-SPRY) domain is indicated, with a conserved signature (WEIDW/C) in the SPRY domain highlighted. Several upstream LRR are also shown. Numbers to the right of the alignment represent the amino acid position of the translated sequences. Click here for file

## References

[B1] Martinon F Tschopp J NLRs join TLRs as innate sensors of pathogens Trends in Immunology 2005 26 8 447 454 10.1016/j.it.2005.06.004 15967716

[B2] Ting JPY Davis BK CATERPILLER: A novel gene family important in immunity, cell death, and diseases Annual Review of Immunology 2005 23 1 387 414 10.1146/annurev.immunol.23.021704.115616 15771576

[B3] Wain HM Lush MJ Ducluzeau F Khodiyar VK Povey S Genew: the Human Gene Nomenclature Database, 2004 updates Nucl Acids Res 2004 32 suppl_1 D255 257 10.1093/nar/gkh072 14681406 PMC308806

[B4] DeYoung BJ Innes RW Plant NBS-LRR proteins in pathogen sensing and host defense Nat Immunol 2006 7 12 1243 1249 10.1038/ni1410 17110940 PMC1973153

[B5] Fritz JH Ferrero RL Philpott DJ Girardin SE Nod-like proteins in immunity, inflammation and disease Nat Immunol 2006 7 12 1250 1257 10.1038/ni1412 17110941

[B6] Kanneganti TD Body-Malapel M Amer A Park JH Whitfield J Franchi L Taraporewala ZF Miller D Patton JT Inohara N Nunez G Critical role for cryopyrin/Nalp3 in activation of caspase-1 in response to viral infection and double-stranded RNA J Biol Chem 2006 281 48 36560 36568 10.1074/jbc.M607594200 17008311

[B7] Ogura Y Sutterwala FS Flavell RA The Inflammasome: First line of the immune response to cell stress Cell 2006 126 4 659 662 10.1016/j.cell.2006.08.002 16923387

[B8] Inohara N Koseki T Lin J del Peso L Lucas PC Chen FF Ogura Y Nunez G An induced proximity model for NF-kappa B activation in the Nod1/RICK and RIP signaling pathways J Biol Chem 2000 275 36 27823 27831 10880512 10.1074/jbc.M003415200

[B9] Ogura Y Inohara N Benito A Chen FF Yamaoka S Nunez G Nod2, a Nod1/Apaf-1 family member that is restricted to monocytes and activates NF-kappa B J Biol Chem 2001 276 7 4812 4818 10.1074/jbc.M008072200 11087742

[B10] Williams KL Lich JD Duncan JA Reed W Rallabhandi P Moore C Kurtz S Coffield VMN Accavitti-Loper MA Su L Vogel SN Braunstein M Ting JPY The CATERPILLER protein monarch-1 is an antagonist of Toll-like Receptor-, tumor necrosis factor-α- , and Mycobacterium tuberculosis-induced pro-inflammatory signals J Biol Chem 2005 280 48 39914 39924 10.1074/jbc.M502820200 16203735 PMC4422647

[B11] Kummer JA Broekhuizen R Everett H Agostini L Kuijk L Martinon F van Bruggen R Tschopp J Inflammasome Components NALP 1 and 3 Show Distinct but Separate Expression Profiles in Human Tissues, Suggesting a Site-specific Role in the Inflammatory Response J Histochem Cytochem 2006 In press (jhc.6A7101.2006) 10.1369/jhc.6A7101.2006 17164409

[B12] Mariathasan S Weiss DS Newton K McBride J O'Rourke K Roose-Girma M Lee WP Weinrauch Y Monack DM Dixit VM Cryopyrin activates the inflammasome in response to toxins and ATP Nature 2006 440 7081 228 232 10.1038/nature04515 16407890

[B13] Chamaillard M Hashimoto M Horie Y Masumoto J Qiu S Saab L Ogura Y Kawasaki A Fukase K Kusumoto S Valvano MA Foster SJ Mak TW Nunez G Inohara N An essential role for NOD1 in host recognition of bacterial peptidoglycan containing diaminopimelic acid Nat Immunol 2003 4 7 702 707 10.1038/ni945 12796777

[B14] Girardin SE Boneca IG Viala J Chamaillard M Labigne A Thomas G Philpott DJ Sansonetti PJ Nod2 is a general sensor of peptidoglycan through muramyl dipeptide (MDP) detection J Biol Chem 2003 278 11 8869 8872 10.1074/jbc.C200651200 12527755

[B15] Hughes AL Evolutionary relationships of vertebrate NACHT domain-containing proteins Immunogenetics 2006 V58 10 785 791 10.1007/s00251-006-0148-8 17006665

[B16] Hibino T Loza-Coll M Messier C Majeske AJ Cohen AH Terwilliger DP Buckley KM Brockton V Nair SV Berney K The immune gene repertoire encoded in the purple sea urchin genome Developmental Biology 2006 300 1 349 365 10.1016/j.ydbio.2006.08.065 17027739

[B17] Inohara N Nunez G Genes with homology to mammalian apoptosis regulators identified in zebrafish. Cell Death Differ 2000 7 5 509 510 10.1038/sj.cdd.4400679 10917738

[B18] Inohara N Koseki T del Peso L Hu Y Yee C Chen S Carrio R Merino J Liu D Ni J Nunez G Nod1, an Apaf-1-like activator of caspase-9 and nuclear factor-kappa B J Biol Chem 1999 274 21 14560 14567 10.1074/jbc.274.21.14560 10329646

[B19] Ogura Y Lala S Xin W Smith E Dowds TA Chen FF Zimmermann E Tretiakova M Cho JH Hart J Greenson JK Keshav S Nunez G Expression of NOD2 in Paneth cells: a possible link to Crohn's ileitis Gut 2003 52 11 1591 1597 10.1136/gut.52.11.1591 14570728 PMC1773866

[B20] Gutierrez O Pipaon C Inohara N Fontalba A Ogura Y Prosper F Nunez G Fernandez-Luna JL Induction of Nod2 in myelomonocytic and intestinal epithelial cells via nuclear factor-kappa B activation J Biol Chem 2002 277 44 41701 41705 10.1074/jbc.M206473200 12194982

[B21] Conti BJ Davis BK Zhang J O'Connor W Jr. Williams KL Ting JPY CATERPILLER 16.2 (CLR16.2), a Novel NBD/LRR family member that negatively regulates T Cell function J Biol Chem 2005 280 18 18375 18385 10.1074/jbc.M413169200 15705585

[B22] Martinon F Agostini L Meylan E Tschopp J Identification of Bacterial Muramyl Dipeptide as Activator of the NALP3/Cryopyrin Inflammasome Current Biology 2004 14 21 1929 10.1016/j.cub.2004.10.027 15530394

[B23] Li X Gold B O'HUigin C Diaz-Griffero F Song B Si Z Li Y Yuan W Stremlau M Mische C Unique features of TRIM5α among closely related human TRIM family members Virology 2007 360 2 419 433 10.1016/j.virol.2006.10.035 17156811

[B24] Chae JJ Wood G Masters SL Richard K Park G Smith BJ Kastner DL The B30.2 domain of pyrin, the familial Mediterranean fever protein, interacts directly with caspase-1 to modulate IL-1beta production PNAS 2006 103 26 9982 9987 10.1073/pnas.0602081103 16785446 PMC1479864

[B25] Song B Gold B O'HUigin C Javanbakht H Li X Stremlau M Winkler C Dean M Sodroski J The B30.2(SPRY) Domain of the Retroviral Restriction Factor TRIM5α Exhibits Lineage-Specific Length and Sequence Variation in Primates J Virol 2005 79 10 6111 6121 10.1128/JVI.79.10.6111-6121.2005 15857996 PMC1091705

[B26] Moretta L Moretta A Killer immunoglobulin-like receptors Current Opinion in Immunology 2004 16 5 626 10.1016/j.coi.2004.07.010 15342010

[B27] Yoder JA Litman RT Mueller MG Desai S Dobrinski KP Montgomery JS Buzzeo MP Ota T Amemiya CT Trede NS Wei S Djeu JY Humphray S Jekosch K Hernandez Prada JA Ostrov DA Litman GW Resolution of the novel immune-type receptor gene cluster in zebrafish PNAS 2004 101 44 15706 15711 10.1073/pnas.0405242101 15496470 PMC524843

[B28] Meyer A Van de Peer Y From 2R to 3R: evidence for a fish-specific genome duplication (FSGD) Bioessays 2005 27 9 937 945 10.1002/bies.20293 16108068

[B29] Dehal P Boore JL Two rounds of whole genome duplication in the ancestral vertebrate PLoS Biol 2005 3 10 e314 10.1371/journal.pbio.0030314 16128622 PMC1197285

[B30] Zhou H Su HS Zhang X Douhan J 3rd Glimcher LH CIITA-dependent and -independent class II MHC expression revealed by a dominant negative mutant J Immunol 1997 158 10 4741 4749 9144488

[B31] Williams KL Taxman DJ Linhoff MW Reed W Ting JPY Cutting Edge: Monarch-1: A pyrin/nucleotide-binding domain/leucine-rich repeat protein that controls classical and nonclassical MHC class I genes J Immunol 2003 170 11 5354 5358 12759408 10.4049/jimmunol.170.11.5354

[B32] Wu CH Apweiler R Bairoch A Natale DA Barker WC Boeckmann B Ferro S Gasteiger E Huang H Lopez R Magrane M Martin MJ Mazumder R O'Donovan C Redaschi N Suzek B The Universal Protein Resource (UniProt): an expanding universe of protein information Nucl Acids Res 2006 34 suppl_1 D187 191 10.1093/nar/gkj161 16381842 PMC1347523

[B33] Ensembl Genome Browser http://www.ensembl.org

[B34] McGinnis S Madden TL BLAST: at the core of a powerful and diverse set of sequence analysis tools Nucl Acids Res 2004 32 suppl_2 W20 25 10.1093/nar/gkh435 15215342 PMC441573

[B35] The Institute for Genomic Research - TIGR http://www.tigr.org

[B36] Kent WJ BLAT---The BLAST-like alignment tool Genome Res 2002 12 4 656 664 10.1101/gr.229202. Article published online before March 2002 11932250 PMC187518

[B37] UCSC Genome Browser http://genome.ucsc.edu

[B38] Marchler-Bauer A Anderson JB Cherukuri PF DeWeese-Scott C Geer LY Gwadz M He S Hurwitz DI Jackson JD Ke Z Lanczycki CJ Liebert CA Liu C Lu F Marchler GH Mullokandov M Shoemaker BA Simonyan V Song JS Thiessen PA Yamashita RA Yin JJ Zhang D Bryant SH CDD: a Conserved Domain Database for protein classification Nucl Acids Res 2005 33 suppl_1 D192 196 15608175 10.1093/nar/gki069 PMC540023

[B39] Schuster-Bockler B Schultz J Rahmann S HMM Logos for visualization of protein families BMC Bioinformatics 2004 5 1 7 10.1186/1471-2105-5-7 14736340 PMC341448

[B40] Kumar S Tamura K Nei M MEGA3: Integrated software for Molecular Evolutionary Genetics Analysis and sequence alignment Brief Bioinform 2004 5 2 150 163 10.1093/bib/5.2.150 15260895

[B41] Thompson JD Higgins DG Gibson TJ CLUSTAL W: improving the sensitivity of progressive multiple sequence alignment through sequence weighting, position-specific gap penalties and weight matrix choice Nucl Acids Res 1994 22 22 4673 4680 10.1093/nar/22.22.4673 7984417 PMC308517

[B42] Purcell MK Kurath G Garver KA Herwig RP Winton JR Quantitative expression profiling of immune response genes in rainbow trout following infectious haematopoietic necrosis virus (IHNV) infection of DNA vaccination Fish and Shellfish Immunology 2004 17 5 447 462 10.1016/j.fsi.2004.04.017 15313511

