# Supplementary material for: A genomic view of the NOD-like receptor family in teleost fish: identification of a novel NLR subfamily in zebrafish
Source: BMC Evol Biol. 2008 Feb 6;8:42. doi: 10.1186/1471-2148-8-42 (PMC2268669; doi:10.1186/1471-2148-8-42)
Supplement: Additional File 1 — Comparison of human and zebrafish NLR-A molecules. Alignment of predicted translations of zebrafish NLR-A genes with their predicted human orthologs. Sequence signatures in the NACHT domains used as boundaries for phylogenetic analyses are highlighted in yellow, and putative leucine-rich repeat motifs (or variants) are highlighted in red. Conserved amino acids between sequences are shown with "*", while ":" and "." represent high or low similarity of amino acids respectively. [file 1471-2148-8-42-S1.doc]

CLUSTAL W (1.83) multiple sequence alignment

DR-NLR-A1 ------------------YLKLLTVHRELLVEQVKNTQCILDNLLMNSFICTEDIEIIQR 42

HS-NOD1 MEEQGHSEMEIIPSESHPHIQLLKSNRELLVTHIRNTQCLVDNLLKNDYFSAEDAEIVCA 60

:::**. :***** :::****::**** *.::.:** **:

DR-NLR-A1 SSTKTDQVRKILELVQSKGEECSAYFTQILHEAYDAYIDLRPWFDEIQYTPLDTIKAIPV 102

HS-NOD1 CPTQPDKVRKILDLVQSKGEEVSEFFLYLLQQLADAYVDLRPWLLEIGFSPSLLTQSKVV 120

..*:.*:*****:******** * :* :*:: ***:*****: ** ::* :: *

DR-NLR-A1 VNTDPISKYCEKLRYELGRDTQFITSYSKSEETPLEDLYTDTQMELLNDTGESLGYLQNL 162

HS-NOD1 VNTDPVSRYTQQLRHHLGRDSKFVLCYAQKEELLLEEIYMDTIMELVGFSNESLGSLNSL 180

*****:*:* ::**:.****::*: .*::.** **::* ** ***:. :.**** *:.*

DR-NLR-A1 DQLLG-DHGVFNPQAETIFIT**GDAGVGKS**IMLQKLQNLWSRRELKTGAKFFFKFRCRAFS 221

HS-NOD1 ACLLDHTTGILNEQGETIFIL**GDAGVGKS**MLLQRLQSLWATGRLDAGVKFFFHFRCRMFS 240

**. *::* *.***** ********::**:**.**: .*.:*.****:**** **

DR-NLR-A1 AFKETDEISLKDLIFKHNCYPDGDPDNEVFAYILRFPETVVFTFDGYDELQMDFDLDNVP 281

HS-NOD1 CFKESDRLCLQDLLFKHYCYPERDPE-EVFAFLLRFPHVALFTFDGLDELHSDLDLSRVP 299

.***:*.:.*:**:*** ***: **: ****::****...:***** ***: *:**..**

DR-NLR-A1 ETVSPEEKTRPLLLLMNLLCGKLLKGSRKILSARSGTEIQSRVIRKKVFLKGFAPEHLKR 341

HS-NOD1 DSSCPWEPAHPLVLLANLLSGKLLKGASKLLTARTGIEVPRQFLRKKVLLRGFSPSHLRA 359

:: .* * ::**:** ***.******: *:*:**:* *: :.:****:*:**:*.**:

DR-NLR-A1 YLALHFPEQEHRMLVSDQLDANPHLCGLCSIPLFSWIILKSFKHLQSVYD-DFELPGSCI 400

HS-NOD1 YARRMFPERALQDRLLSQLEANPNLCSLCSVPLFCWIIFRCFQHFRAAFEGSPQLPDCTM 419

* ***: : : .**:***:**.***:***.***::.*:*:::.:: . :**.. :

DR-NLR-A1 TLTNVFLLLSEVFLGHSTARPGLLRRTLRCPTETFKAGEQKLSGFARLALHGIETSKLVF 460

HS-NOD1 TLTDVFLLVTEVHLNRMQPSS-LVQRNTRSPVETLHAGRDTLCSLGQVAHRGMEKSLFVF 478

***:****::**.*.: . . *::*. *.*.**::**.:.*..:.::* :*:*.* :**

DR-NLR-A1 TLDEAVCCGLNDEDLQFGFLRPASHYDSS-SASSFEFLHETLQAFLAAFSLVLDAKLNPE 519

HS-NOD1 TQEEVQASGLQERDMQLGFLRALPELGPGGDQQSYEFFHLTLQAFFTAFFLVLDDRVGTQ 538

* :*. ..**::.*:*:****. .. ... . .*:**:* *****::** **** ::..:

DR-NLR-A1 SILKFFSKCKYKK-SSRLSCIP------CLKNTKPRESDAFQTN--FQFTNLFLCGLLSK 570

HS-NOD1 ELLRFFQEWMPPAGAATTSCYPPFLPFQCLQGSGPAREDLFKNKDHFQFTNLFLCGLLSK 598

.:*:**.: :: ** * **:.: * ..* *:.: **************

DR-NLR-A1 SNAALLEHLVPPSALKQKRKILKSYLSNSVKTHLKSLPRSPSTDIEGDKVHAMPNFLWML 630

HS-NOD1 AKQKLLRHLVPAAALRRKRKALWAHLFSSLRGYLKSLPRVQVESFN--QVQAMPTFIWML 656

:: **.****.:**::*** * ::* .*:: :****** .:: :*:***.*:***

DR-NLR-A1 RCIFETNSEDVAKMTANGISADYIKIAFCNIYSADCSALNFVLHHRRKHLGVDMDNNNIN 690

HS-NOD1 RCIYETQSQKVGQLAARGICANYLKLTYCNACSADCSALSFVLHHFPKRLALDLDNNNLN 716

***:**:*:.*.:::*.**.*:*:*:::** *******.***** *:*.:*:****:*

DR-NLR-A1 DYGVKQLRPSFSKMTVVRFCVNQLTDSGIEVLAEELIRYKIVKVLGIHSLYQNHITDVGA 750

HS-NOD1 DYGVRELQPCFSRLTVLRLSVNQITDGGVKVLSEELTKYKIVTYLG---LYNNQITDVGA 773

****::*:*.**::**:*:.***:**.*::**:*** :****. **: **:*:******

DR-NLR-A1 KQVAKIIEECPHLRTVKLGCNNITSVGGKYLASAIHKSKSIFDIGMWGNCIGDEGAEAFA 810

HS-NOD1 RYVTKILDECKGLTHLKLGKNKITSEGGKYLALAVKNSKSISEVGMWGNQVGDEGAKAFA 833

: *:**::** * :*** *:*** ****** *:::**** ::***** :*****:***

DR-NLR-A1 EALKNHPSLTNLSLSANGITSHGGRSLAQTLKENTSLHIVWLIQNKISDDAASDLAEAFR 870

HS-NOD1 EALRNHPSLTTLSLASNGISTEGGKSLARALQQNTSLEILWLTQNELNDEVAESLAEMLK 893

***:******.***::***::.**:***::*::****.*:** **::.*:.*..*** ::

DR-NLR-A1 SNSSLTHFYRLIDNEFTIDGARQLSEGLKDNTTLKEV----------------------- 907

HS-NOD1 VNQTLKHLW-LIQNQITAKGTAQLADALQSNTGITEICLNGNLIKPEEAKVYEDEKRIIC 952

*.:*.*:: **:*::* .*: **::.*:.** :.*:

DR-NLR-A1 -

HS-NOD1 F 953

CLUSTAL W (1.83) multiple sequence alignment

DR-NLR-A2 ---------------------------MNAQQLILKQRAELLAVLCGGGSDEPLESVLDL 33

HS-NLR2 MGEEGGSASHDEEERASVLLGHSPGCEMCSQEAFQAQRSQLVELLVSG-SLEGFESVLDW 59

* :*: : **::*: :* .* * * :*****

DR-NLR-A2 LLAQEVLVWEDYLRVRVAEKPLCANIRQLLDLVYDKGEDACSYFLAAIEQELAEEQKAGL 93

HS-NLR2 LLSWEVLSWEDYEGFHLLGQPLSHLARRLLDTVWNKGTWACQKLIAAAQEAQADSQSPKL 119

**: *** **** .:: :**. *:*** *::** **. ::** :: *:.*.. *

DR-NLR-A2 CFGNGCVMVGKDRPATATSTLLADRPMLVRRLRDNIDGALNILLTTGCFSIKDCDSVQLP 153

HS-NLR2 HG------CWDPHSLHPARDLQSHRPAIVRRLHSHVENMLDLAWERGFVSQYECDEIRLP 173

:: . :. .: * :.** :****:.:::. *:: * .* :**.::**

DR-NLR-A2 VYTPSQQVRRLLDQVKFKGETAAKTLLEYLEQPEPTSPISAEKENTPSADCLVYQKKLRS 213

HS-NLR2 IFTPSQRARRLLDLATVKANGLAAFLLQHVQELPVPLALPLE-----AATCKKYMAKLRT 228

::****:.***** ...*.: * **::::: . .:. * :* * * ***:

DR-NLR-A2 SVASQSLFLSTYGGTGRFSLDDIYTDGHLEVMNSSGET-------TTLGLEDVVGPMGTL 266

HS-NLR2 TVSAQSRFLSTYDGAETLCLEDIYTENVLEVWADVGMAGPPQKSPATLGLEELFSTPGHL 288

:*::** *****.*: :.*:****:. *** . * : :*****::... * *

DR-NLR-A2 NEDADTVLVS**GEAGSGKS**TLVQRLHLLWAREALLLNTFLLFPFSCRKLNAEHRELSLKEL 326

HS-NLR2 NDDADTVLVV**GEAGSGKS**TLLQRLHLLWAAGQDFQEFLFVFPFSCRQLQCMAKPLSVRTL 348

*:******* **********:******** : : :::******:*:. : **:: *

DR-NLR-A2 LFLHCCWPDRNQDEVFQFILDHPHLVLFTFDGLDEFKLGFTDEERHCCPTKQVPIPVLLF 386

HS-NLR2 LFEHCCWPDVGQEDIFQLLLDHPDRVLLTFDGFDEFKFRFTDRERHCSPTDPTSVQTLLF 408

** ****** .*:::**::****. **:****:****: ***.****.**. ..: .***

DR-NLR-A2 NLLQGTLMKGVMKVVTSRPHAVGPSLKRYLRKEVLLKGFSPGGIDCFVKKHYSDPAMATR 446

HS-NLR2 NLLQGNLLKNARKVVTSRPAAVSAFLRKYIRTEFNLKGFSEQGIELYLRKRHHEPGVADR 468

*****.*:*.. ******* **.. *::*:*.*. ***** **: :::*:: :*.:* *

DR-NLR-A2 VIESVQGNTALLGLCHIPVFCWIVIKCYQELLAGQDGIPQTITDVYLLVLQHFFQRKSS- 505

HS-NLR2 LIRLLQETSALHGLCHLPVFSWMVSKCHQELLLQEGGSPKTTTDMYLLILQHFLLHATPP 528

:*. :* .:** ****:***.*:* **:**** :.* *:* **:***:****: : :.

DR-NLR-A2 -QPQSGLGKAWLAEHLDTVLKLGELALEGLQTSCYVFSGYELQRNRITEQDVGIGFLIYC 564

HS-NLR2 DSASQGLGPSLLRGRLPTLLHLGRLALWGLGMCCYVFSAQQLQAAQVSPDDISLGFLVRA 588

....*** : * :* *:*:**.*** ** .*****. :** ::: :*:.:***: .

DR-NLR-A2 SDISVNDCKRYEFLHITLQCFFAALYVILNRNNDRSAISRLFQPRNRQVSGLSQSCLGQC 624

HS-NLR2 KGVVPGSTAPLEFLHITFQCFFAAFYLALSADVPPALLRHLFNCGRPGNSPMARLLPTMC 648

..: .. ******:******:*: *. : : : :**: . * ::: *

DR-NLR-A2 MDHSVEES-------HEAETANLQITAQFVSGLLSQRHNNLLLECCP-AAVRERNVKQVV 676

HS-NLR2 IQASEGKDSSVAALLQKAEPHNLQITAAFLAGLLSREHWGLLAECQTSEKALLRRQACAR 708

:: * :. ::**. ****** *::****:.* .** ** . . *. .

DR-NLR-A2 KSLSKRMQRHFKSIPRPVEGEKKSMHAMPSFVWLIKCIYELQDNSIAQDAMAKLDVEHLK 736

HS-NLR2 WCLARSLRKHFHSIPPAAPGEAKSVHAMPGFIWLIRSLYEMQEERLARKAARGLNVGHLK 768

.*:: :::**:*** .. ** **:****.*:***:.:**:*:: :*:.* *:* ***

DR-NLR-A2 LTYCNIGPVECTALAYVLKNLRKPVGLQLDNNSVGDVGVEQLLPCLPMCHSLYLRNNNIS 796

HS-NLR2 LTFCSVGPTECAALAFVLQHLRRPVALQLDYNSVGDIGVEQLLPCLGVCKALYLRDNNIS 828

**:*.:**.**:***:**::**:**.**** *****:********* :*::****:****

DR-NLR-A2 DEGIRKLLEKGIECENFQKIALFNNKLTDTCTQYFSCLLKSKQNFLALRLGNNNITSVGA 856

HS-NLR2 DRGICKLIECALHCEQLQKLALFNNKLTDGCAHSMAKLLACRQNFLALRLGNNYITAAGA 888

*.** **:* .:.**::**:********* *:: :: ** .:*********** **:.**

DR-NLR-A2 EQLAEGLSYNQSLQFLGLWGNKVGDRGAEVLADALTNSKTLIWLSLVDNGVGSAGACALA 916

HS-NLR2 QVLAEGLRGNTSLQFLGFWGNRVGDEGAQALAEALGDHQSLRWLSLVGNNIGSVGAQALA 948

: ***** * ******:***:***.**:.**:** : ::* *****.*.:**.** ***

DR-NLR-A2 KFIRQNKSLEELWLNKNSICKEGVDCLIEALKMNTSVK---------------------- 954

HS-NLR2 LMLAKNVMLEELCLEENHLQDEGVCSLAEGLKKNSSLKILKLSNNCITYLGAEALLQALE 1008

:: :* **** *::* : .*** .* *.** *:*:*

DR-NLR-A2 --------------------------------

HS-NLR2 RNDTILEVWLRGNTFSLEEVDKLGCRDTRLLL 1040

CLUSTAL W (1.83) multiple sequence alignment

DR-NLR-A3 MDRHSQIPWQEYCYLDLKSNAPKQETSKRMEDHGWLRRHQPLLHRFLSTSILVNVVHQMR 60

HS-NOD3 MEMDAPRPP----SLAVPGAASR---PGRLLDGGHGRQQ----VQALSSQLLEVIPDSMR 49

*: .: * * : . *.: . *: * * *:: :: : **:.:* : ..**

DR-NLR-A3 KADLLSTQEAGLIQEMGSLKDKVHLLVEVLSVSDPHGAALQAYLQSSYQTEYNLITIHDS 120

HS-NOD3 KQEVRTGREAGQGHGTGSPAEQVKALMDLLAGKGSQGSHAPQALDRTPDAPLGPCS-NDS 108

* :: : :*** : ** ::*: *:::*: ...:*: *: : :: . :::**

DR-NLR-A3 VVQPYKDTLLQWLKEDENSLAQVAGSSQGFT-ILLIESVSDLQQREHDLVQVSVNRGAGP 179

HS-NOD3 RIQRHRKALLSKVGGG----PELGGPWHRLASLLLVEGLTDLQLREHDFTQVEATRGGG- 163

:* ::.:**. : . :.::.*. : :: :**:*.::*** ****:.**...**.*

DR-NLR-A3 LHGRALGLDKLIAPLTRVSTAPRVTLTV**GVAGSGKS**RMVSRFIRLWSSGQIYPELSLAIP 239

HS-NOD3 HPARTVALDRLFLPLSRVSVPPRVSITI**GVAGMGKT**TLVRHFVRLWAHGQVGKDFSLVLP 223

.*::.**:*: **:***..***::*:**** **: :* :*:***: **: ::**.:*

DR-NLR-A3 IACWELSSYDRISVERLLRLFVPYDNMDVIIFNESCKVLLILDGLEEFRQTLDFADAPPT 299

HS-NOD3 LTFRDLNTHEKLCADRLICSVFPHVGEPSLAVAVPARALLILDGLDECRTPLDFSNTVAC 283

:: :*.:::::..:**: ..*: . : . ..:.*******:* * .***::: .

DR-NLR-A3 SDPRREIPVSDLIANIVRGNLLPGATLWLLSRPGIGAKVPAGLVDRVTEAPPLSQTQKSS 359

HS-NOD3 TDPKKEIPVDHLITNIIRGNLFPEVSIWITSRPSASGQIPGGLVDRMTEIRGFNEEEIKV 343

:**::****..**:**:****:* .::*: ***. ..::*.*****:** :.: : .

DR-NLR-A3 SSQTDIPQDLSSQVWTY--LNSQKLLLILSSVPAICHVIVTTLSRLIKIES---EATPLP 414

HS-NOD3 CLEQMFPEDQALLGWMLSQVQADRALYLMCTVPAFCRLTGMALGHLWRSRTGPQDAELWP 403

. : :*:* : * ::::: * ::.:***:*:: :*.:* : .: :* *

DR-NLR-A3 -RTLTEVYAHYCWPHLSKS------------DTASGIRKSLNTLGRLAFYSLLRRRYTFV 461

HS-NOD3 PRTLCELYSWYFRMALSGEGQEKGKASPRIEQVAHGGRKMVGTLGRLAFHGLLKKKYVFY 463

*** *:*: * ** . :.* * ** :.*******:.**:::*.*

DR-NLR-A3 ESELRTYGVDVPPQVGTLSYRILQRKQSWSSDNKAWQFTHTSVQEFLGALYYYVSSRRAV 521

HS-NOD3 EQDMKAFGVDLALLQGAPCSCFLQREETLAS-SVAYCFTHLSLQEFVAAAYYYGASRRAI 522

*.:::::***:. *: . :***::: :* . *: *** *:***:.* *** :****:

DR-NLR-A3 FDLFSESGVSWPRIGFHSHYRAALQKTNQSDATLQGSLDLFMRFLSGLLSPTAGVLLGGP 581

HS-NOD3 FDLFTESGVSWPRLGFLTHFRSAAQRAMQAED---GRLDVFLRFLSGLLSPRVNALLAGS 579

****:********:** :*:*:* *:: *:: : * **:*:********* ...**.*.

DR-NLR-A3 LGVSREEQVTQRTTAMTLLQNAVAAAGGDAVSMRSVNMVTCLAELQQGEWLRSIEEDLIS 641

HS-NOD3 L-LAQGEHQAYRTQVAELLQGCLRPD--AAVCARAINVLHCLHELQHTELARSVEEAMES 636

* ::: *: : ** . ***..: . **. *::*:: ** ***: * **:** : *

DR-NLR-A3 CGLRGKLKGGVCAVLAYLLLVSDSCTEETQLSNCLDSSSLKRLLPQLLYCSKLRMENNEF 701

HS-NOD3 GALARLTGPAHRAALAYLLQVSDACAQEANLSLSLSQGVLQSLLPQLLYCRKLRLDTNQF 696

.* . *.***** ***:*::*::** .*... *: ******** ***::.*:*

DR-NLR-A3 KEGAMELLGSLLSAKECHIQMLSLADNSISSKGVKPLSRALLVNRTLTTLDLRGNNIGAK 761

HS-NOD3 QDPVMELLGSVLSGKDCRIQKISLAENQISNKGAKALARSLLVNRSLTSLDLRGNSIGPQ 756

:: .******:**.*:*:** :***:*.**.**.*.*:*:*****:**:******.**.:

DR-NLR-A3 GAKTLCEALKLNQFLVSVNLQNNHIEDEGARSLSEVLQSNRKLTTLNVQKNGIGPA-LKK 820

HS-NOD3 GAKALADALKINRTLTSLSLQGNTVRDDGARSMAEALASNRTLSMLHLQKNSIGPMGAQR 816

***:*.:***:*: *.*:.**.* :.*:****::*.* ***.*: *::***.*** ::

DR-NLR-A3 IAEGLIKNQTLQDFNVSSNHLGDLGTVALAQALMVNHVLHTLSLQSNSVSDRGIKALSHA 880

HS-NOD3 MADALKQNRSLKELMFSSNSIGDGGAKALAEALKVNQGLESLDLQSNSISDAGVAALMGA 876

:*:.* :*::*::: .*** :** *: ***:** **: *.:*.*****:** *: ** *

DR-NLR-A3 LQSNRGLCCLNLRENSIGVAGAKDIAKALKVNTCLRELDLTANLLHDEGVTAIAEAMRVN 940

HS-NOD3 LCTNQTLLSLSLRENSISPEGAQAIAHALCANSTLKNLDLTANLLHDQGARAIAVAVREN 936

* :*: * .*.******. **: **:** .*: *::**********:*. *** *:* *

DR-NLR-A3 RSISSLHNIQQWNFMKAGAAKALAESLSSNTCIQLLDLQENALGDNGVIALAAALMSNSS 1000

HS-NOD3 RTLTSLH--LQWNFIQAGAAQALGQALQLNRSLTSLDLQENAIGDDGACAVARALKVNTA 994

*:::*** : ****::****:**.::*. * .: *******:**:*. *:* ** *::

DR-NLR-A3 LTVLYLQGVSAGKSGAVALADAMVVNKTLHTLDLRGNSIGMEGAKAFSSALKNNRSLRSL 1060

HS-NOD3 LTALYLQVASIGASGAQVLGEALAVNRTLEILDLRGNAIGVAGAKALANALKVNSSLRRL 1054

**.**** .* * *** .*.:*:.**:**. ******:**: ****::.*** * *** *

DR-NLR-A3 NLQENSLGMDGAIFIATALRGNHQLTYINLQGNGIGESGAKVVSDAIKAGAPDCVVDI 1118

HS-NOD3 NLQENSLGMDGAICIATALSGNHRLQHINLQGNHIGDSGARMISEAIKTNAPTCTVEM 1112

************* ***** ***:* :****** **:***:::*:***:.** *.*::

CLUSTAL W (1.83) multiple sequence alignment

DR-NLR-A4 MERMDWSGDALTLVAQEASVLVDLLCEQDSSVLDHIFDLLESNTQDEIRSLINNRDRVSA 60

HS-NOD4 -----MDPVGLQLGNKNLWSCLVRLLTKDPEWLNAKMKFFLPNTDLDSRNETLDPEQRVI 55

: : . .* * :: : * :*.. *: :.:: .**: : *. : ::

DR-NLR-A4 IVDYFKTSDNITCRKFFCTIYEYCKDIPFYLETTLVSIAG-------------------- 100

HS-NOD4 LQLNKLHVQGSDTWQSFIHCVCMQLEVPLDLEVLLLSTFGYDDGFTSQLGAEGKSQPESQ 115

: :. : * ::*: **. *:* *

DR-NLR-A4 --DPIDTNFPLTDNYSSSRNVKRLRLDPLQSYTDALKSMVKEKHKTVMQSVVKDIRLDDT 158

HS-NOD4 LHHGLKRPHQSCGSSPRRKQCKKQQLELAKKYLQLLRTSAQQRYRSQIPGSGQPHAFHQV 175

. :. . .. . :: *: :*: :.* : *:: .:::::: : . : :.:.

DR-NLR-A4 WVYLKHRNPPRAKDR-TLQLQASLDSHEGEESEHKVSVESLLKTTGRVVVLL**GQAGSGKT** 217

HS-NOD4 YVPPILRRATASLDTPEGAIMGDVKVEDGADVSISDLFNTRVNKGPRVTVLL**GKAGMGKT** 235

:* *... : * : ..:. .:* : . . .:: ::. **.****:** ***

DR-NLR-A4 LLVHCLGHSWAENTFPSIQLLFLLEFRQLNLISRDLSLKELLFLFYPACSYVEEEKEEVF 277

HS-NOD4 TLAHRLCQKWAEGHLNCFQALFLFEFRQLNLITRFLTPSELLFDLYLSP---ESDHDTVF 292

*.* * :.***. : .:* ***:********:* *: .**** :* : :*.::: **

DR-NLR-A4 AFILTHPDKVCFILDGYDEFRAKLTDPRELESTVDLSTPMPMADLLSGLCSRKILPDCTV 337

HS-NOD4 QYLEKNADQVLLIFDGLDEALQPMGP----------DGPGPVLTLFSHLCNGTLLPGCRV 342

:: .:.*:* :*:** ** : : : :. * *: *:* **. .:**.* *

DR-NLR-A4 LVTCRPRDVTDMFGSPGLLTCELQGFDRLGVKEYAEQYFHEKGDLKTNAVNLLMDNRHLL 397

HS-NOD4 MATSRPGKLPACLPAEAAMVHMLG-FDGPRVEEYVNHFFSAQ-PSREGALVELQTNGRLR 400

:.*.** .:. : : . :. * ** *:**.:::* : : .*: * * :*

DR-NLR-A4 SMSHVPGLCHICCICVDYLFSSGE-VLNHQLPTSLTQIYIQILLAFLSRFNEDGVSKTSL 456

HS-NOD4 SLCAVPALCQVACLCLHHLLPDHAPGQSVALLPNMTQLYMQMVLALSPPGHLP------- 453

*:. **.**::.*:*:.:*:.. . * ..:**:*:*::**: . : : :

DR-NLR-A4 LMRYRTKIVEMSQLALKGLEGSTIVFMESEVSKELQDFGVRSGILSRVKLTHEDGSSGYG 516

HS-NOD4 ----TSSLLDLGEVALRGLETGKVIFYAKDIAPPLIAFGATHSLLTSFCVCTGPGHQQTG 509

:: :.::::.::**:*** ..::* .::: * **. .:*: . : * . *

DR-NLR-A4 FTFMHLTMQEFLAALHLMTSQTITDGQLKKKLNLKTRWTTKTDPKSVFTDSVHLYVCGLA 576

HS-NOD4 YAFTHLSLQEFLAALHLMASPKVNKDTLTQYVTLHSRWVQRTKARLGLSDHLPTFLAGLA 569

::* **::**********:* .:... *.: :.*::**. :*..: ::* : ::.***

DR-NLR-A4 AEACTSSLIQLKGAESEMAWVKKRQAAVRNVLLGLAGGASHTGPKIVELCRCAHETQDAK 636

HS-NOD4 SCTCRPFLSHLAQGN--EDCVGAKQAAVVQVLKKLAT-RKLTGPKVVELCHCVDETQEPE 626

: :* . * :* .: * :**** :** ** . ****:****:*..***:.:

DR-NLR-A4 LAKAIGSRP--QIELRNIRLNAVDMDSLAFVTAAADQTVCLDFGGCSIDLDCLEILPSFK 694

HS-NOD4 LASLTAQSLPYQLPFHNFPLTCTDLATLTNILEHREAPIHLDFDGCPLEPHCPEALVGCG 686

**. .. *: ::*: *...*: :*: : : .: ***.**.:: .* * * .

DR-NLR-A4 NVDHLIFRSRKYDDKFAETLCGILSKLQALQQLDFISGGLTDVGAAKLAKALEDCPNITH 754

HS-NOD4 QIENLSFKSRKCGDAFAEALSRSLPTMGRLQMLGLAGSKITARGISHLVKALPLCPQLKE 746

::::* *:*** .* ***:*. *..: ** *.: .. :* * ::*.*** **::..

DR-NLR-A4 LNVSDNSLKDEGIREIAETVSRLHNISSILMGKNNISTDGILTLIERMAACTSV-----Q 809

HS-NOD4 VSFRDNQLSDQVVLNIVEVLPHLPRLRKLDLSSNSICVSTLLCLARVAVTCPTVRMLQAR 806

:.. **.*.*: : :*.*.:.:* .: .: :..*.*... :* * . .:*.:* :

DR-NLR-A4 EVHVEYVHLLACQIHFELKSLIKIQSKSGMYIVSVMFSFQILFHSWFLKMRDTFLFIDFL 869

HS-NOD4 ERTIIFLLSPPTETTAELQRAPDLQESDGQRKGAQSRSLTLRLQKCQLQVHDAEALIALL 866

* : :: . : **: .:*...* : *: : ::. *:::*: :* :*

DR-NLR-A4 KDKDIIIFILMQCSHLFD------CFSLCTGEIKRSTLASQNALTQS------GEFSKDW 917

HS-NOD4 QEGPHLEEVDLSGNQLEDEGCRLMAEAASQLHIARKLDLSDNGLSVAGVHCVLRAVSACW 926

:: : : :. .:* * . : . .* *. *:*.*: : .* *

DR-NLR-A4 LFKELVIPQISKLCYSFLH----------FLFAHLLWLVLHLASDCSAMLFILNLKQCNL 967

HS-NOD4 TLAELHISLQHKTVIFMFAQEPEEQKGPQERAAFLDSLMLQMPSELPLSSRRMRLTHCGL 986

: ** *. * :: *.* *:*::.*: . :.*.:*.*

DR-NLR-A4 TVSHVASLCNKLRGFAIL--TLLDLSNNSLGNKGLKKILDLLPKLGGIQEINVSENGVDM 1025

HS-NOD4 QEKHLEQLCKALGGSCHLGHLHLDFSGNALGDEGAARLAQLLPGLGALQSLNLSENGLSL 1046

.*: .**: * * . * **:*.*:**::* :: :*** **.:*.:*:****:.:

DR-NLR-A4 EGVVMLASALCKQYNLKRVDASENGKKKVVLGFDTSRDCEGGISLPQHPQTS-LLSVTQN 1084

HS-NOD4 DAVLGLVRCFSTLQWLFRLDISFESQHILLRGDKTSRDMWATGSLPDFPAAAKFLGFRQR 1106

:.*: *. .:.. * *:* * :.:: :: * .**** . ***:.* :: :*.. *.

DR-NLR-A4 PRPPFISLTHSDIQPAYMTKLCNQLVKCKNLLDIDFSNLKLTDDSVENLMLILPDMLSLH 1144

HS-NOD4 CIPRSLCLSECPLEPPSLTRLCATLKDCPGPLELQLSCEFLSDQSLETLLDCLPQLPQLS 1166

* :.*:.. ::*. :*:** * .* . *::::* *:*:*:*.*: **:: .*

DR-NLR-A4 VLNLSHVDLSTNGALILVRSLADCQRVTSVELRYFMVHTQHYYTNKNHLLTLCHVNRLTE 1204

HS-NOD4 LLQLSQTGLSPKSPFLLANTLSLCPRVKKVDLRSLHHATLHFRSNEEEEGVCCGR--FTG 1224

:*:**:..**.:..::*..:*: * **..*:** : * *: :*::. . * :*

DR-NLR-A4 YNLSKTNVVKLSKILESCPHLSELDLSHNVLRDEGVKCFVDHLPKLKISSSVSLIGNRMT 1264

HS-NOD4 CSLSQEHVESLCWLLSKCKDLSQVDLSANLLGDSGLRCLLECLPQVPISGLLDLSHNSIS 1284

.**: :* .*. :*..* .**::*** *:* *.*::*::: **:: **. :.* * ::

DR-NLR-A4 QIGALHLVNSMNTCEKVVAVEVSLGVEDQSLIQFVQEHANGKTLRLRECSFELAHLKMLV 1324

HS-NOD4 QESALYLLETLPSCPRVREASVNLGSEQSFRIHFSREDQAGKTLRLSECSFRPEHVSRLA 1344

* .**:*:::: :* :* ..*.** *:. *:* :*. ****** ****. *:. *.

DR-NLR-A4 KILTKCPRLLSLELSSNSLNSQGLFALLDSLAELSTIQTLTIEEPWMTGSAAIGLIARCL 1384

HS-NOD4 TGLSKSLQLTELTLTQCCLGQKQLAILLSLVGRPAGLFSLRVQEPWADRARVLSLLEVCA 1404

. *:*. :* .* *:. .*..: * **. :.. : : :* ::*** : .:.*: *

DR-NLR-A4 SLQQHIREIRISETILKDLQSISDLSFPRKKIQNLRKEEEKECEGDTGREENTIAGRDVE 1444

HS-NOD4 QASGSVTEISISETQQ---QLCVQLEFPRQ-EENPEAVALRLAHCDLGAHHSLLVGQLME 1460

. . : ** **** :* :*.***: :* . : .. * * ... :.*: :*

DR-NLR-A4 EQD-FLLLSSTMIKKRPVPKTPDILINTFFCSLSEN--------IVTDQAGEKLLTALSH 1495

HS-NOD4 TCARLQQLSLSQVNLCEDDDASSLLLQSLLLSLSELKTFRLTSSCVSTEGLAHLASGLGH 1520

: ** : :: .:..:*::::: **** *: :. :* :.*.*

DR-NLR-A4 CRALQQLNLSENQFGPQGSSVLCEGLVSMKALKKLQMHFTIPDS----ILKIQYIQRICK 1551

HS-NOD4 CHHLEELDLSNNQFDEEGTKALMRALEGKWMLKRLDLSHLLLNSSTLALLTHRLSQMTCL 1580

*: *::*:**:***. :*:..* ..* . **:*:: . : :* :*. : * *

DR-NLR-A4 YSIKLFLTSVESSDLVGVASSLKHCPSIEDVSLSWNGCGDSLARTLAEILPLCTKLKRLD 1611

HS-NOD4 QSLRLNRNSIGDVGCCHLSEALRAATSLEELDLSHNQIGDAGVQHLATILPGLPELRKID 1640

*::* .*: . . ::.:*: ..*:*::.** * **: .: ** *** .:*:::*

DR-NLR-A4 LEANNITTAGATLIAKCLPLCPSIE----------------------------------- 1636

HS-NOD4 LSGNSISSAGGVQLAESLVLCRRLEELMLGCNALGDPTALGLAQELPQHLRVLHLPFSHL 1700

*..*.*::**.. :*:.* ** :*

DR-NLR-A4 ------------------------------------------------------------

HS-NOD4 GPGGALSLAQALDGSPHLEEISLAENNLAGGVLRFCMELPLLRQIDLVSCKIDNQTAKLL 1760

DR-NLR-A4 ------------------------------------------------------------

HS-NOD4 TSSFTSCPALEVILLSWNLLGDEAAAELAQVLPKMGRLKRVDLEKNQITALGAWLLAEGL 1820

DR-NLR-A4 ----------------------------------------------

HS-NOD4 AQGSSIQVIRLWNNPIPCDMAQHLKSQEPRLDFAFFDNQPQAPWGT 1866

CLUSTAL W (1.83) multiple sequence alignment

DR-NLR-A5 MWRFGRPSGSEVLKWRCQRSSRDTCCKLHTMALQKTVNLSLQSAGICRNIKRCHLSFPIK 60

HS-NOD5 -MRWGHHLPRASWGSGFRRALQRPDDRIPFLIHWSWPLQGERPFGPPRAFIRHHGSSVDS 59

: *:*: :*: : . :: : . . :. * * : * * * .

DR-NLR-A5 TASSCRNAWKKQLTPMCYSASGFNHRLFCTSQPVTDPIEIHRQKLALWFSHIPQEERQFS 120

HS-NOD5 APPSGR------------------HGRLFPSASATEAIQRHRRNLAEWFSRLPREERQFG 101

:..* * : : : * : .* ..*:.*: **::** ***::*:*****.

DR-NLR-A5 GYVSPETMHVEPLILERHPDEDR---GLSPFKEKTSVCAQQSLTVGDLFHDSNHGKARGL 177

HS-NOD5 PTFALDTVHVDPVIRESTPDELLRPPAELALEHQPPQAGLPPLALSQLFNPDACGR-RVQ 160

.: :*:**:*:* * *** . .::.:.. .. .*::.:**: . *: *

DR-NLR-A5 NILMY**GAVGTGKS**TVIRKLVQDWCDGSLFSQFKLMLPFSCEDISQLSKA-ISLRDLVGRK 236

HS-NOD5 TVVLY**GTVGTGKS**TLVRKMVLDWCYG-RLPAFELLIPFSCEDLSSLGPAPASLCQLVAQR 219

.:::**:*******::**:* *** * :. *:*::******:*.*. * ** :**.::

DR-NLR-A5 YMHLRKTPYLSGENDMARDVLFIFHGMEKMKLDFRISSTELCSDPNEALLSGAIVVNLLR 296

HS-NOD5 YTPLKEVLPLMAA--AGSHLLFVLHGLEHLNLDFRLAGTGLCSDPEEPQEPAAIIVNLLR 277

* *::. * . . .:**::**:*:::****::.* *****:*. ..**:*****

DR-NLR-A5 KYMLPEASILVTTRLSAVDRVPKKYINRYVQICGFNDLDRQRAYFTSRLLQQHEG----- 351

HS-NOD5 KYMLPQASILVTTRPSAIGRIPSKYVGRYGEICGFSDTNLQKLYFQLRLNQPYCGYAVGG 337

*****:******** **:.*:*.**:.** :****.* : *: ** ** * : *

DR-NLR-A5 ----KPDRAAEMLIEMLYLNLQRESQLATACFLPSYCWLTCATLHLLHFTDTKSPIRTLT 407

HS-NOD5 SGVSATPAQRDHLVQMLSRNLEGHHQIAAACFLPSYCWLVCATLHFLHAPTPAG--QTLT 395

. : *::** **: . *:*:**********.*****:** . . . ::***

DR-NLR-A5 GIYTSFMRLNFGGEVITLNGGVSSQEQQNSLMLYVVRTVGKLAFDGITNKRTSFTAEELE 467

HS-NOD5 SIYTSFLRLNFSGETLDS-----TDPSNLSLMAYAARTMGKLAYEGVSSRKTYFSEEDVC 450

.*****:****.**.: : :: .: *** *..**:****::*::.::* *: *::

DR-NLR-A5 QWVGGKTKTDEELRELAVFRTDVLDFFLVPRDDYGSDPETKGRRYVFAVPIMQEYLAALY 527

HS-NOD5 GCLEAGIRTEEEFQLLHIFRRDALRFFLAPCVEPG-----RAGTFVFTVPAMQEYLAALY 505

: . :*:**:: * :** *.* ***.* : * :. :**:** *********

DR-NLR-A5 VVLGENKTVLEKLTKEVSEALGPASEDITSLLSVLSKIIPFRIF-AIFNLLKLFPKLFEK 586

HS-NOD5 IVLGLRKTTLQKVGKEVAELVGRVGEDVSLVLGIMAKLLPLRALPLLFNLIKVVPRVFGR 565

:*** .**.*:*: ***:* :* ..**:: :*.:::*::*:* : :***:*:.*::* :

DR-NLR-A5 VSSYSKGRIANTMAAEMFRSEDSFNEDVLDQVEQSLLGVHGP--QPQEEIYTRAFELYPI 644

HS-NOD5 MVGKSREAVAQAMVLEMFREEDYYNDDVLDQMGASILGVEGPRRHPDEPPEDEVFELFPM 625

: . *: :*::*. ****.** :*:*****: *:***.** :*:* ..***:*:

DR-NLR-A5 FMGGLLHYGNRRLLDQLGCNIKSHTVSQITRALRKNLIKQSQKNQPPEELMDLLVLLYEF 704

HS-NOD5 FMGGLLSAHNRAVLAQLGCPIKNLDALENAQAIKKKLGKLGRQVLPPSELLDHLFFHYEF 685

****** ** :* **** **. . : ::*::*:* * .:: **.**:* *.: ***

DR-NLR-A5 QNPRLTAEVLQSIKSLNLSTVRMTPHKCFVLSTVLGCTTSSFHLNELNLSSCHITPELLQ 764

HS-NOD5 QNQRFSAEVLSSLRQLNLAGVRMTPVKCTVVAAVLGS--GRHALDEVNLASCQLDPAGLR 743

** *::****.*::.***: ***** ** *:::***. . . *:*:**:**:: * *:

DR-NLR-A5 MLWPAFRHTRNLNLQFNSLDPESCILLRDLLLEPNCTIKSLQLCDNYLSDSGISHLLEAL 824

HS-NOD5 TLLPVFLRARKLGLQLNSLGPEACKDLRDLLLHDQCQITTLRLSNNPLTEAGVAVLMEGL 803

* *.* ::*:*.**:***.**:* ******. :* *.:*:*.:* *:::*:: *:*.*

DR-NLR-A5 SGNHSLQRLNLMHTGLSDKSALLLAEKLGQHDQLKELNVAYNNIGDSAALTLVDACREHP 884

HS-NOD5 AGNTSVTHLSLLHTGLGDEGLELLAAQLDRNRQLQELNVAYNGAGDTAALALARAAREHP 863

:** *: :*.*:****.*:. *** :*.:: **:*******. **:***:*. *.****

DR-NLR-A5 SIHTVHLYLNQLTDVAKQSLYVRGGPRVKEGRRVKVLASVTEGSDISEDWHPILSVIGKN 944

HS-NOD5 SLELLHLYFNELSSEGRQVLRDLGG--AAEGG-ARVVVSLTEGTAVSEYWSVILSEVQRN 920

*:. :***:*:*:. .:* * ** . ** .:*:.*:***: :** * *** : :*

DR-NLR-A5 SLSWERDRVREQLLVFLKDLEWGRKQHLSFWKKMHFRRVESVVRQMLRFIEKSSDTGTGT 1004

HS-NOD5 LNSWDRARVQRHLELLLRDLEDSRGATLNPWRKAQLLRVEGEVRALLEQLGSSGS----- 975

**:* **:.:* ::*:*** .* *. *:* :: ***. ** :*. : .*..

DR-NLR-A5 K 1005

HS-NOD5 -
