# Supplementary material for: A genomic view of the NOD-like receptor family in teleost fish: identification of a novel NLR subfamily in zebrafish
Source: BMC Evol Biol. 2008 Feb 6;8:42. doi: 10.1186/1471-2148-8-42 (PMC2268669; doi:10.1186/1471-2148-8-42)
Supplement: Additional File 2 — Tables describing gene predictions and their locations for NLR-A, -B and -C genes in zebrafish genome assembly version 7. [file 1471-2148-8-42-S2.doc]

xNxL LxxLxLxxNxL LxxLxLxxN

NLRC57 SCKLTVQCCESLSSVLQSSNCVLRELDLSNNDLQDSGVKLLSDGLKSQHCKLDTLRLQSC 716

TC359229_1 SCNLTVQCCESLSSALQSSNCVLRELDLSNNDLQDSGVKKLSDALKSQQCKLETLR---- 109

TC363713_2 MCNLTAQSCETFSSVLQSSNCVLRELDLSNNDLQDSGVELLSDGLKSQHCKLETLRLAMC 121

*:**.*.**::**.***********************: ***.****:***:***:

xL LxxLxLxxNxL LxxLxL

NLRC57 NLTVQCCESLSSALQSSNCVLRELDLSNNDLQDSGVKKLSAGLKKP-----ECKLEILRL 771

TC359229_1 -----------------------------------------------------------L 110

TC363713_2 NLSAQSCESLSSVLQSSNCVLRELDLSNNDLQDSGVKKLSDGLKSLRVLGENCKLEKLRL 181

: : : :: : : : : : : : : *

xxNxL LxxLxLxxNxL

NLRC57 SGCMVTEEGCGFLSSALTSNPSHLRELDLSYNHPGDSGVKLLSEQLEDPNYTLDKLNLDH 831

TC359229_1 SGCMVTEEGCGFLSSALTSNPSHLRELDLSYNHPGDSGVKLLSEQLEDPNYTLNKLNLDH 170

TC363713_2 SGCMVTEEGCGFLSSALTSNPSHLRELDLSYNHPGDSRVKLLSEQLEDPNYTLDKLNLDH 241

************************************* ***************:******

PRY>>>>>>>>>>>>>>>>>>>>>>>>>>>>>>>>>>>>>>>>>>>>

NLRC57 GGDTRITAGPHKYVCFLTLDPNTAHTHLILSEENREVKHVRENQPYPDHPDRFDVYP--Q 889

TC359229_1 GGDKRITAGPRKYVCFLTLDPNTAHTKLILSEENRELKSVRENQPYPDHPDRFDGYYCLQ 230

TC363713_2 GGEKRITAGLHKYVCFLTLDPNTTNTQLILSEKNREVKNVRENQPYPDHPDRFDYYS--Q 299

**:.***** :************::*:*****:***:* *************** * *

>>>>>>>SPRY###############################################

NLRC57 VLCRESVCGRCY**WEIDW**SG-DHGVCISVSYTSIRRKGRGDECWFGYNAQSWSLICSPSRF 948

TC359229_1 VLCRESVCGRCY**WEIDW**SGEDHGVEISVSYKSIRRKRR---------------------- 268

TC363713_2 VLCRESVCGRCY**WEIDC**SG-DGVVDISVSYKSIRRKGGGVE------------------- 339

**************** ** * * *****.*****
