# Supplementary material for: A genomic view of the NOD-like receptor family in teleost fish: identification of a novel NLR subfamily in zebrafish
Source: BMC Evol Biol. 2008 Feb 6;8:42. doi: 10.1186/1471-2148-8-42 (PMC2268669; doi:10.1186/1471-2148-8-42)
Supplement: Additional File 3 — The B30.2 domain of zebrafish NLR-C. Alignment of zebrafish NLR-C57 (see Table 4) with similar cDNA sequences identified in the TIGR database. The B30.2 (PRY-SPRY) domain is indicated, with a conserved signature (WEIDW/C) in the SPRY domain highlighted. Several upstream LRR are also shown. Numbers to the right of the alignment represent the amino acid position of the translated sequences. [file 1471-2148-8-42-S3.doc]

Supplementary Table 1

NLR-SUBFAMILY A in zebrafish genome version 7

| **Nickname** | **Chromosome (or scaffold)** | **Location** | **ENSEMBL prediction ID** | **REFSEQ/**  **uniprot ID** |
| --- | --- | --- | --- | --- |
| NLR-A1/NOD1 | (Zv7_NA3145) | 13.9 k | ENSDARG00000036308 | XP_699379 |
| NLR-A2/NOD2 | 7 | 25.9 m | ENSDARG00000010756 | **Q1AMZ9** |
| NLR-A3/NOD3 | 24 | 34.9 m | ENSDARG00000061564 | XP_697414 |
| NLR-A4/NOD4 | 18 | 16.5 m | ENSDARG00000024631 |  |
| NLR-A5/NOD5 | 15 | 22.7 m | ENSDARESTG00000003051 | XP_685481 |

Supplementary Table 2

NLR-SUBFAMILY B in zebrafish genome version 7

| **Nickname** | **Chromosome** | **Location (m)** | **ENSEMBL prediction ID**  **(**ENSDARG000000) | **REFSEQ ID** |
| --- | --- | --- | --- | --- |
| NLR-B1 | 2 | 39.15 |  | XP_694951 |
| NLR-B2 | 2 | 39.17 | 38493 | XP_694893 |
| NLR-B3 | 2 | 41.18 | 04582 |  |
| NLR-B4 | 2 | 43.54 | 38549 |  |
| NLR-B5 | - | - | Gene prediction removed |  |
| NLR-B6 | 15 | 39.62 | 41744 |  |

Supplementary Table 3

NLR-SUBFAMILY C in zebrafish genome version 7 (selected examples)

| **Nickname** | **Chromosome (or scaffold)** | **Location** | **ENSEMBL prediction ID**  **(**ENSDARG000000) | **REFSEQ ID** |
| --- | --- | --- | --- | --- |
| NLR-C1 | 3 | 49.0 m |  | XP_692844 |
| NLR-C2 | (Zv7_2527) | 3.2 k | 52239 |  |
| NLR-C3 | 25 | 32.6 m | 57488 | XP_688571 |
| NLR-C4 | 6 | 33.7 m | 54997 |  |
| NLR-C5 | (Zv7_2487) | 259 k | 54809 |  |
| NLR-C6 | - | - | Gene prediction removed |  |
| NLR-C7 | 4 | 36.1 m | 54521 | XP_689993 |
| NLR-C8 | 4 | 36.4 m | 54506 |  |
| NLR-C9 | 4 | 37.7 m |  | XP_698595 |
| NLR-C10 | 4 | 33.1 m | 54412 |  |
| NLR-C11 | 12 | 42.0 m |  | XP_692726 |
| NLR-C12 | - | - | Gene prediction removed |  |
| NLR-C13 | - | - | Gene prediction removed |  |
| NLR-C14 | - | - | Gene prediction removed |  |
| NLR-C15 | (Zv7_2508) | 179 k | 53299 |  |
| NLR-C16 | - | - | Gene prediction removed |  |
| NLR-C17 | - | - | Gene prediction removed |  |
| NLR-C18 | 4 | 33.5 m |  | XP_686111 |
| NLR-C19 | 6 | 39.1 m | 52946 |  |
| NLR-C20 | 12 | 41.3 m | 52760 | XP_686816 |
| NLR-C21 | 12 | 41.9 m | 52752 | XP_689021 |
| NLR-C22 | - | - | Gene prediction removed |  |
| NLR-C23 | 4 | 34.0 m | 52776 |  |
| NLR-C24 | 14 | 28.3 m | 55661 |  |
| NLR-C25 | 14 | 29.3 m | 71537 | XP_693637 |
| NLR-C26 | - | - | Gene prediction removed |  |
| NLR-C27 | 14 | 29.9 m |  | XP_694145 |
| NLR-C28 | 3 | 35.1 m |  | XP_693652 |
| NLR-C29 | (Zv7_2561) | 1.0 m | 54752 |  |
| NLR-C30 | (Zv7_2487) | 259 k | 54465 |  |
| NLR-C31 | 14 | 31.9 m |  | XP_691478 |
| NLR-C32 | - | - | Gene prediction removed |  |
| NLR-C33 | 14 | 33.2 m | 53848 | XP_689023 |
| NLR-C34 | - | - | Gene prediction removed |  |
| NLR-C35 | 16 | 51.7 m | 53051 | XP_698503 |
| NLR-C36 | 17 | 6.9 m |  | XP_690676 |
| NLR-C37 | 17 | 7.7 m |  | XP_689954 |
| NLR-C38 | 18 | 28.0 m | 56197 | XP_693868 |
| NLR-C39 | 20 | 6.3 m | 43785 |  |
| NLR-C40 | - | - | Gene prediction removed |  |
| NLR-C41 | 22 | 26.5 m | 55030 |  |
| NLR-C42 | 6 | 33.7 m | 55004 |  |
| NLR-C43 | - | - | Gene prediction removed |  |
| NLR-C44 | 22 | 38.0 m |  | XP_688350 |
| NLR-C45 | 22 | 59.3 m |  | XP_697815 |
| NLR-C46 | (Zv7_2558) | 25.0 k | 54149 | XP_687258 |
| NLR-C47 | (Zv7_2558) | 19.4 k |  | XP_690327 |
| NLR-C48 | 23 | 42.2 m | 39900 |  |
| NLR-C49 | 8 | 13.3 m | 53964 |  |
| NLR-C50 | (Zv7_2497) | 69.4 k |  | XP_687591 |
| NLR-C51 | - | - | Gene prediction removed |  |
| NLR-C52 | - | - | Gene prediction removed |  |
| NLR-C53 | (Zv7_2525) | 179.3 k | 55178 | XP_691896 |
| NLR-C54 | 18 | 28.1 m | 55023 |  |
| NLR-C55 | Unknown |  |  | XP_689864 |
| NLR-C56 | Unknown |  |  | XP_690860 |
| NLR-C57 | Unknown |  |  | XP_694605 |
| NLR-C58 | 1 | 53.5 |  | XP_694871 |
| NLR-C59 | Unknown |  |  | XP_696432 |
| NLR-C60 | Unknown |  |  | XP_698814 |
| NLR-C61 | Unknown |  |  | XP_699128 |
| NLR-C62 | Unknown |  |  | XP_697396 |
